# 5-Substituted 4-Thiouridines, 4-Thio-2′-deoxyuridines and Their Oligoglycol Carbonate Prodrugs as Promising Antimicrobial Agents

**DOI:** 10.3390/ijms262311712

**Published:** 2025-12-03

**Authors:** Dmitry A. Makarov, Maxim V. Jasko, Sergey D. Negrya, Inna L. Karpenko, Elizabeth V. Urbina, Vladimir O. Chekhov, Olga V. Efremenkova, Byazilya F. Vasilyeva, Danila V. Zimenkov, Anastasia I. Ushtanit, Sergey N. Kochetkov, Liudmila A. Alexandrova

**Affiliations:** 1 Engelhardt Institute of Molecular Biology, Russian Academy of Sciences, 119991 Moscow, Russia; dmitmakarov_97@mail.ru (D.A.M.); z@biochip.ru (D.V.Z.);; 2 Gause Institute of New Antibiotics, 119021 Moscow, Russia

**Keywords:** nucleosides, 5-substituted 4-thionucleosides, antibacterial activity, *mycobacteria*

## Abstract

The problem of antibiotic resistance is one of the challenges that science and medicine face in the 21st century. Nucleoside analogs have already proven as antiviral and antitumor agents, and, currently, there are more and more reports on their antibacterial and antifungal activity. The substitution of an oxygen atom by a sulfur one leads to the emergence of unique properties. Here, we report the synthesis of eight new 4-thioanalogs of 5-substituted (5-alkyloxymethyl and 5-alkyltriazolylmethyl) derivatives of 2′-deoxyuridine and uridine, which were active against *Mycobacterium tuberculosis* and Gram-positive bacteria. The novel sulfur-containing nucleosides were synthesized via activation of the pyrimidine C4 position, followed by condensation with thioacetic acid and deblocking. To increase the solubility, oligoglycol carbonate depot forms were obtained via activation of the 3′-hydroxyl group using *N*,*N*’-carbonyldiimidazole and condensation with triethylene glycol. The highest inhibitory activity was demonstrated by 3′-triethylene glycol depot forms of 4-thio-5-undecyl- and 5-dodecyloxymethyl-2′-deoxyuridine (**4a**,**b**) against two strains of *M. smegmatis*. The most promising compounds were 5-[4-decyl-(1,2,3-triazol-1-yl)methyl]-4-thio-2′-deoxy- and ribouridine (**3c**,**g**) and 5-undecyloxymethyl 4-thiouridine (**3e**) active toward clinical *M. intracellulare* isolates. Overall, novel sulfur-containing nucleoside analogs were low toxic, demonstrated better inhibitory activity compared to their C4-oxo ones, and, thus, are promising compounds for the development of new antibacterial agents.

## 1. Introduction

The discovery of antibiotics in the 20th century was a revolutionary event in human history, which saved millions of human lives [1,2,3]. Today, the overuse of antibiotics has led to the emergence of antibiotic resistance, giving priority to drug-resistant strains [4,5]. Inappropriate prescribing practices, increasing self-medication, and non-compliance to treatment are leading to an accelerating spread of resistant pathogens [6] that cannot be eradicated by standard chemotherapy regimens, thus causing increased mortality from infectious diseases. The World Health Organization (WHO) has declared antimicrobial resistance one of the top 10 health problems affecting humanity [6,7,8,9].

Emerging since the 1990s, the multidrug-resistant strains of *Mycobacterium tuberculosis* pose a particular danger [10,11,12]. According to the WHO 2050 forecast, about a quarter of the 10 million deaths associated with drug resistance will be caused by drug-resistant tuberculosis strains (TB) [10]. Globally, in 2022, TB caused an estimated 1.40 million deaths [13]. In the European Region, the incidence rate decreased from 42 per 100,000 population in 2010 to 24 in 2022 [13]. While the incidence of tuberculosis has declined in developed countries, reporting data on non-tuberculous infections and diseases has shown a steady upward trend [14]. For example, in Australia, the estimated incidence rate increased from 11 per 100,000 in 2001 to 26 per 100,000 population in 2016 [15]. Nontuberculous infections are associated with age and various immunocompromised states [16,17,18]. *Mycobacteria* are found in patients with bronchiectasis [19,20] and cystic fibrosis [21]. They are the confirmed agents of nosocomial infections after surgery interventions [22] and even less traumatic cosmetic procedures [23]. *Mycobacterium* is a diverse genus, which includes more than 400 species, and 246 of them are associated with pathological processes in humans [24].

Many strategies have been proposed to combat antibiotic resistance; one of them is introducing into medical practice new antibacterial drugs that are selective for new strains and aimed at new targets [25,26].

Nucleoside derivatives are attractive compounds for the development of drugs based on them. They have already proven to act as antiviral [27] and antitumor agents [28], and, currently, there are more and more reports on the antibacterial (including antituberculosis) and antifungal activities of such compounds [29,30,31,32,33,34,35,36,37].

Modified nucleosides, nucleotides, and oligonucleotides with an oxygen atom replaced by chalcogen atoms (sulfur, selenium, tellurium) are of great interest for scientific research. For example, the 5-substituted 2-sulfur- and selenouridines are natural components of the bacterial tRNA epitranscriptome [38]. The substitution of an oxygen atom in some components of nucleic acids by chalcogen atoms leads to the emergence of unique properties in such biomolecules associated with their altered physical and chemical properties. Sulfur-containing nucleosides occupy a special place among modified nucleosides [39]. Sulfur-containing functional groups are prevailing components found in various pharmacologically active substances and some natural products. They are valuable in the field of medicinal chemistry and are included in a large number of approved drugs and clinical candidates [40,41,42,43,44]. Previous studies have demonstrated that the introduction of a sulfur atom into a nucleoside molecule can lead to a significant increase in their antiviral [45], anticancer [39], and antituberculosis activity [37,46] compared to oxygen-containing analogs.

Previously, we obtained 5-substituted derivatives of 2′-deoxyuridine and uridine [47,48], which showed significant inhibitory activity against Gram-positive bacteria, including drug-resistant strains of *M. tuberculosis* and *S. aureus*. At present, the exact mechanism of their action remains unclear. However, our studies have shown that one of the possible targets of action of nucleoside derivatives may have direct or indirect effects on the bacterial cell wall [49,50]. On the other hand, we demonstrated that the mechanism of action of 5-alkyloxymethyl-2′-deoxyuridine 5′-monophosphates may be partially associated with inhibition of the flavin-dependent thymidylate synthase ThyX [51].

**Figure 1:**
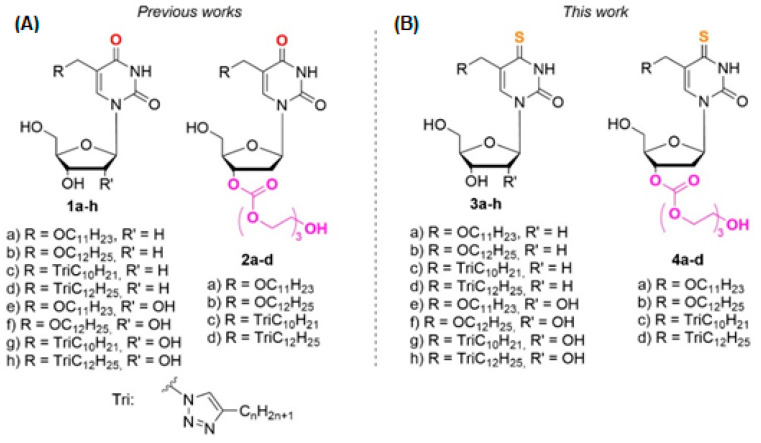
Modified derivatives of pyrimidine nucleosides: (**A**) **1a**–**h** and **2a**–**d**, synthesized earlier, (**B**) C4-thio analogs **3a**–**h** and **4a**–**d** presented in this work.

The aim of this work is to synthesize C4-thio derivatives of 2′-deoxyuridine and uridine containing an extended lipophilic fragment at the 5-position (Figure 1) and to study their antibacterial activity.

Also, based on the fact that an oxygen-sulfur substitution at the C4 position of nitrogenous base should increase the lipophilicity of modified nucleosides **3a**–**h** and decrease the solubility in aqueous media, we obtained their oligoglycol carbonate prodrugs **4a**–**h**. As we have shown earlier, this approach allowed us to increase the solubility of 5-modified 2′-deoxyuridines by at least 2 orders of magnitude, while maintaining their antibacterial activity [52,53,54]. The results of enzymatic hydrolysis and theoretical computations, reported in our previous work [53], make it possible to assume with sufficient certainty that our synthetic carbonyltriethylene glycol derivatives of modified nucleosides are depot forms of the nucleosides possessing antibacterial activity. Moreover, in the same work, it was shown that the enzymatic hydrolysis half-time for the corresponding 4-oxo- and 4-thioderivatives did not differ significantly (τ_1/2_ (h) = 3 for **2b** and τ_1/2_ (h) = 6 for **4a**).

## 2. Results

### 2.1. Chemistry

**Scheme 1:**
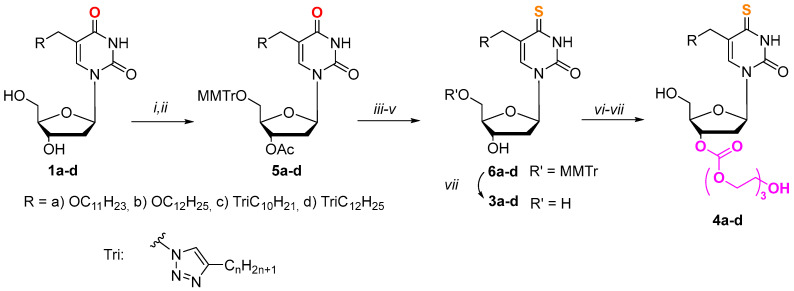
Synthesis of derivatives **3a**–**d** and **4a**–**d**. (**i**) MMTrCl (1.5 eq.), pyridine, 37 °C, 12 h; (**ii**) Ac_2_O (1.5 eq.), pyridine, r.t., 12 h; (**iii**) 1,2,4-triazole (6 eq.), 2-chlorophenyl dichlorophosphate (2.2 eq.), pyridine, r.t., 24 h; (**iv**) AcSH (1.5 eq.), pyridine, dioxane, r.t., 20 h; (**v**) NH_3_/H_2_O, EtOH, r.t., 12 h; (**vi**) (1) CDI (4 eq.), DMF, 37 °C, 24 h, (2) triethylene glycol (20 eq.), dioxane, 37 °C, 24 h; (**vii**) AcOH, H_2_O, r.t., 12 h.

To obtain 4-thio derivatives of 5-alkyloxymethyl-2′-deoxyuridines, we initially intended to use a reaction with Lawesson’s reagent [55], but it did not lead to the formation of the expected compounds. Compounds **3a**–**d** were synthesized according to Scheme 1, starting from 5-alkyloxymethyl-2′-deoxyuridine **1a**,**b** [47] and 5-alkyltriazolylmethyl-2′-deoxyuridine **1c**,**d** [47] on a mmol scale using a combination of different protecting groups. For further obtaining their 3′-triethyleneglycol depot forms, derivatives **5a**–**d** were synthesized (Scheme 1), containing an acid-labile monomethoxytrityl fragment at the 5′-position and an acetyl group at the 3′-position. Thus, a monomethoxytrityl protective group at the 5′-position of the carbohydrate fragment was introduced at the first stage. Further treatment with the acetic anhydride allowed us to obtain 3′,5′-protected derivatives **5a**–**d**.

The replacement of the oxygen atom by a sulfur one in the pyrimidine fragment in the case of 2′-deoxyuridine derivatives (Scheme 1) was carried out through activation of the C4 position according to the method of Divakar and Reese [56] in a later modification [57], followed by condensation with thioacetic acid (AcSH). To obtain derivatives **6a**–**d,** the acetyl protecting group was removed. The 3′-hydroxyl group was activated using *N*,*N*’-carbonyldiimidazole (CDI), and condensation with triethylene glycol was carried out according to our previously developed method [52,54]. The target compounds **4a**–**d** were synthesized by treating the condensation products with acetic acid.

**Scheme 2:**
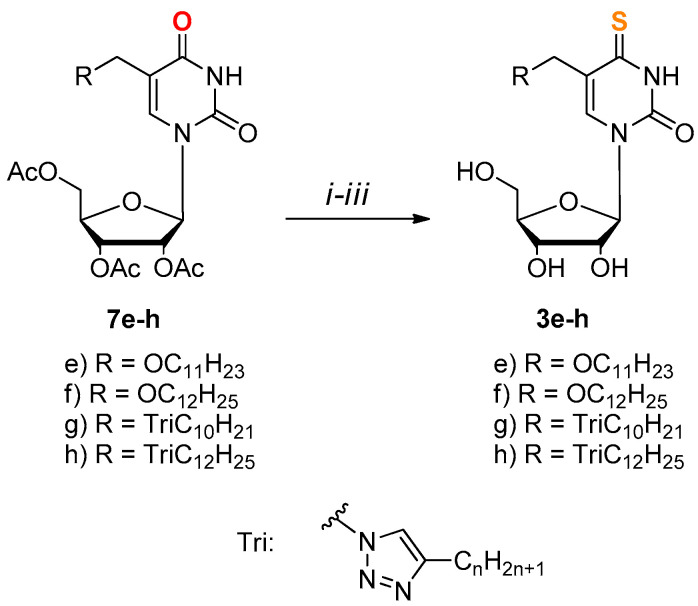
Synthesis of derivatives **3e**–**h**. (**i**) SOCl_2_, (1.6 eq.) DMF, DCE, Δ, 6 h; (**ii**) AcSH, (1.5 eq.), pyridine, dioxane, r.t., 12 h; (**iii**) NH_3_/H_2_O, EtOH, r.t., 12 h.

Compounds **3e**–**h** were prepared from protected nucleosides **7e**–**h** by condensation with thioacetic acid via 4-chloro derivatives obtained, according to the Žemlička and Šorm method [58,59] (Scheme 2).

The purity and structure of the target compounds were confirmed by ^1^H- and ^13^C-NMR spectroscopy and high-resolution mass spectrometry (see the Supplementary Materials).

### 2.2. Water Solubility

As we initially assumed, an oxygen-sulfur substitution at the C4 position of the nitrogenous base of synthesized compounds has led to a decrease in their water solubility by at least 1.5 times compared with corresponding 4-oxoanalogs [52,54]. For example, 0.013 mg/mL for the 4-thioderivative **3a** and 0.017 mg/mL for the derivative **1a**. Other oxygen-containing derivatives showed solubility in the range of 0.005–0.028 mg/mL for 2′-deoxynucleosides **1a**–**d**, 0.03–0.1 mg/mL for ribonucleosides **1e**–**h**, and 0.78–3.6 mg/mL for oligoglycol carbonate prodrugs **2a**–**d**.

The introduction of the oligoglycol carbonate fragment into the target nucleoside molecules allowed us to increase the solubility of compounds **3a**–**d** by at least an order of magnitude (Table 1).

### 2.3. Cytotoxicity

The cytotoxicity of the synthesized compounds (CD_50_) was estimated by MTT assay [60] in *HeLa* and *A549* cell lines. The compounds demonstrated cytotoxic activity at concentrations of 30–60 μM (**3a**, **3b**, **3e**, **3f**, **4a**, **4b**) and 70–100 μM (**3c**, **3d**, **3g**, **3h**, **4c**, **4d**) in both cell lines. For most compounds, the toxicity turned out to be approximately the same as for previously synthesized 4-oxo-derivatives. The exact cytotoxicity values for each compound are shown in Table S1 (see the Supplementary Materials).

### 2.4. In Vitro Study of Antibacterial Activity of the Obtained Compounds

Antibacterial effect of the obtained compounds was studied by their ability to suppress the growth of a number of microorganisms in vitro using two different methods: (1) bacterial strains from the collection of the Gause Institute of New Antibiotics were tested using the same method as in our previous studies [48]; and (2) mycobacterial strains and clinical isolates from the collection of the EIMB were studied using a previously developed method [61]. The details of testing bacteria for susceptibility to the developed compounds are described in the Materials and Methods Section.

The antibacterial activities were tested against the following Gram-positive bacteria: two strains of *Staphylococcus aureus* (ESKAPE group), including methicillin-resistant strain MRSA (MRSA strains occur widely and cause nosocomial infections that resist the modern antibiotic therapy) and *Micrococcus luteus* NCTC 8340, and mycobacteria: two strains of *Mycobacterium smegmatis* (which are used for the preliminary assessment of the activity followed by the analysis of promising compounds against the strains of the causative agent of tuberculosis, i.e., *Mycobacterium tuberculosis*). Some compounds under investigation showed activity against Gram-positive bacteria as well as mycobacteria (Table 2). The highest inhibitory activity (5 and 21 mg/L, respectively) was demonstrated by 3′-triethylene glycol depot forms of 5-undecyl- and 5-dodecyloxymethyl-2′-deoxyuridine (**4a**,**b**, respectively) against two strains of *M. smegmatis*, in contrast to the corresponding 4-oxo derivatives **2a**,**b**, which were less active against the same strains (24 and 25 mg/L). These same derivatives also exhibited inhibitory action at concentrations of 5 and 21 mg/L, respectively, for strains of *S. aureus* and *Mic. luteus*. Unfortunately, compounds **4c**,**d** and ribo derivatives **3e**–**h** were inactive against the above-mentioned microorganisms. Apparently, the low solubility of the studied compounds **1a**–**d**, **3a**–**d** did not allow achieving inhibitory concentrations.

### 2.5. In Vitro Study of Antimycobacterial Activity of the Obtained Compounds

The biological activity of the developed compounds toward various clinical isolates of *Mycobacterium* (both slow- and rapidly growing species) was tested (Table 3) as in [61].

The MIC of compounds **3a**, **3b**, **3d**, and **3h,** as well as oxo derivatives **1a**–**h,** was ≥80 mg/L, which was above the maximal tested concentration, and thus, no antimycobacterial activity was found. Compounds **2a**–**d** have not been tested against these mycobacterial strains. 4-Thio derivatives **3c**, **3e**, **3g**, **4a**, **4b**, and **4c** showed noticeable activity toward all tested isolates of slow-growing species, while **3f** and **4d** showed variable MICs in the range of 10 mg/L to more than 80 mg/L, which corresponded to the maximal tested concentration. All developed oligoglycol carbonate prodrugs **4a**–**h** demonstrated activity against *M. avium* (20–80 mg/L), with the lowest MIC of 20 mg/L for compound **4c**. The lowest MIC of 10 mg/L was recorded for **3f**, **3e**, and **4a** compounds against the slow-growing *M. intracellulare* isolates. Compound **3e** was also active toward less-susceptible fast-growing *M. abscessus*, *M. fortuitum,* and slow-growing *M. avium*, while low MICs of 20 mg/L and ≥80 mg/L were recorded for different isolates. *M. fortuitum* also had an MIC of 80 mg/L for compound **3g**. Other compounds were not active toward fast-growing strains and isolates of mycobacteria (Table 3). The details of testing *mycobacteria* for susceptibility to the developed compounds are described in the Materials and Methods Section.

## 3. Discussion

**Figure 2:**
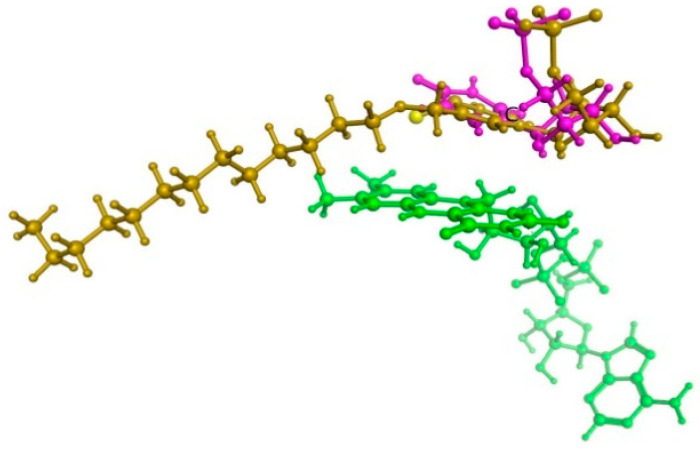
The superposition of dUMP substrate (marked purple) and 5-dodecyloxymethyl-4-thio-2′-deoxyuridine 5′-monophosphate (yellow) within ThyX active site. Flavine cofactor fragment (green) is also shown.

For further research into microbial inhibitors, rational methods for the synthesis of C4-thio derivatives of 2′-deoxyuridine and uridine containing an extended lipophilic fragment at the 5-position were developed. We assumed that replacing the oxygen atom in the C4 position of the nitrogenous base with a sulfur atom could lead to increased antibacterial properties of such derivatives. A hypothetical ThyX enzyme inhibitor, 5-dodecyloxymethyl-4-thio-2′-deoxyuridine 5′-monophosphate, was docked in a binding site of *M. tuberculosis* ThyX (PDB file 2AF6) [62] using the Molecular Operating Environment program (MOE) [63], as in our previous work [51]. The calculations revealed no significant differences in the binding of 5-alkyloxymethyl-4-thio-2′-deoxyuridine 5′-monophosphate. Figure 2 illustrates that the 4-thio inhibitor of ThyX and substrate (dUMP) have the same binding mode. The structure of 5-dodecyloxymethyl-4-thio-dUMP-ThyX complex (obtained in silico) and amino acid residues in contact with 5-dodecyloxymethyl-4-thio-dUMP are shown in Supplementary Figures S1 and S2.

To obtain 4-thio derivatives of 5-modified 2′-deoxyuridines, a reaction with Lawesson’s reagent was first carried out [55]. This reagent allows direct one-step thionylation of the starting nucleosides [64,65]. However, in the case of 5-alkyloxymethyl derivatives, the reaction proceeded with the formation of a product of unknown structure. Similar unusual reactions with both Lawesson’s reagent and phosphorus pentasulfide are known in the literature, in each case involving the removal of a group located in the benzylic position in the presence of a carbonyl oxygen atom [66,67]. Expected derivatives were obtained through activation of the C4 position using either the method of Divakar and Reese [56,57] or the method of Žemlička and Šorm [58,59], followed by condensation with thioacetic acid and deprotection. It has been shown that Šorm‘s reaction can be a good alternative to the Divakar and Reese method. Both methods lead to the production of thio derivatives in approximately the same yields, but the first one is easier to perform. To increase the solubility of 5-modified 4-thio-2′-deoxyuridines, we used a previously developed prodrug approach, namely the introduction of triethyleneglycol fragment into the 3′-position of modified nucleosides [52,53,54].

The novel sulfur-containing nucleoside analogs demonstrated better inhibitory activity (MIC 5–80 mg/L) compared to their C4-oxo ones, and were low-toxic. The highest inhibitory activity (5 and 21 mg/L, respectively) was demonstrated by 3′-triethylene glycol depot forms of 4-thio-5-undecyl- and 5-dodecyloxymethyl-2′-deoxyuridine (**4a**,**b**, respectively) against two strains of *M. smegmatis*, in contrast to the corresponding 4-oxo derivatives.

**Figure 3:**
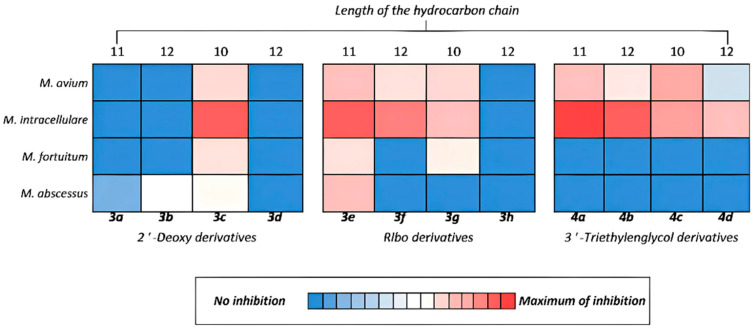
Inhibitory activity towards mycobacteria of synthesized derivatives **3a**–**h** and **4a**–**d**.

The greatest activity against mycobacteria was shown by both ribo- and 2′-deoxyribonucleosides containing a triazolyl fragment with an alkyl chain length of 10 carbon atoms (derivatives **3c**, **3g**) and derivative **3e** containing an undecyloxymethyl substituent (Figure 3). This can be explained by the fact that derivatives with this size of hydrocarbon substituent exhibit the best solubility in aqueous media (Table 1). Since a dependence was observed, the larger the hydrocarbon substituent (and therefore the lower the solubility), the less activity the compounds exhibited (Figure 3). Furthermore, unlike 5-alkyloxymethyl derivatives (**3a**, **3e**, **4a**), triazole derivatives (**3c**, **3d**, **3g**, **3h**, **4c**, **4d**) are capable of improving the binding of the resulting compounds to biological targets due to the formation of additional hydrogen bonds and dipole interactions [68,69]. Ribonucleosides (**3e**–**h**) (containing an additional hydroxyl group at the 2′-position of the carbohydrate residue) or 2′-deoxyribonucleosides with a triethylene glycol moiety at the 3′-position (**4a**–**d**) generally inhibited mycobacterial growth better than the corresponding 2′-deoxy derivative. These results can be explained by the better solubility of the studied compounds compared to ones that did not show activity.

Thus, the substitution of an oxygen atom in the C4 position of 5-modified nucleosides by a sulfur one leads to a significant increase in their antibacterial activity compared to oxygen-containing analogs. This is most clearly demonstrated in the case of inhibition of non-tuberculous mycobacteria: inactive oxo derivatives are converted into effective inhibitors of mycobacterial growth. They can serve as promising lead compounds for the development of new antibacterial (primarily antimycobacterial) agents. Overall, sulfur-containing analogs of modified nucleosides appear to be valuable lead compounds for the development of new therapeutic agents.

## 4. Materials and Methods

### 4.1. Chemistry

Commercial reagents from Fluka (Buchs, Switzerland), Sigma-Aldrich (St. Louis, MO, USA), and Acros Organics (Geel, Belgium) were used in this work. Solvents were purified using standard methods. Column chromatography was performed using Kieselgel 60 (40–63 μm) silica gel (Merck, Darmstadt, Germany). ^1^H and ^13^C NMR spectra were recorded with a Bruker AM300 at ambient temperature in DMSO-*d*_6_ and CDCl_3_ solutions. Chemical shift values are given in δ scale relative to Me_4_Si. The *J* values are given in hertz. UV spectra were recorded on a spectrophotometer SF-102 (Akvilon, Moscow, Russia) in methanol. High-resolution mass spectra were recorded on a micrOTOF-Q II device (Bruker Daltonics, Bremen, Germany) by electrospray ionization mass spectrometry (ESI-MS). Measurements were carried out in two ion modes: positive and negative. The following parameters were used: capillary voltage 4500 V; mass scanning range: for positive ion polarity *m*/*z* 50–2500, for negaitive ion polarity *m*/*z* 50–3000; external calibration with LC/MS Calibration standard for ESI-TOF (Agilent Technologies Inc., Santa Clara, CA, USA); gas pressure 0.4 bar; nitrogen spray gas (4 L/min); interface temperature: 180 °C; and flow rate 200 μL/min. Samples were injected into the mass-spectrometer chamber from an HPLC system Agilent 1260 (Agilent Technologies Inc., Santa Clara, CA, USA) equipped with an Agilent Poroshell 120 EC-C18 column (3.0 × 50 mm; 2.7 μm) (Agilent Technologies Inc., Santa Clara, CA, USA). The samples were eluted in the acetonitrile gradient in 0.1% formic acid with a flow rate of 200 μL/min. Molecular ions in the spectra were analyzed and matched with the appropriately calculated m/z and isotopic profiles in the Bruker Data Analysis 4.0 program. Dry samples were dissolved in 100% methanol. All reactions were monitored with thin-layer chromatography (TLC) and carried out with Merck (Darmstadt, Germany) precoated plates DCAlufolienKieselgel60 F_254_.

#### Study of Water Solubility of Synthesized Compounds

To evaluate the solubility, a mixture of 20 mg of a compound and 3 mL of water was stirred on a magnetic stirrer for 24 h. The pellet was removed by centrifugation (14,000 rpm, 10 min). The compound concentration was inferred from UV absorption of the resulting solution.

### 4.2. General Method for the Synthesis of 3′-O-Acetyl-5′-O-Monomethoxytrityl-5-Alkyloxymethyl- and 5-[4-Alkyl-(1,2,3-Triazol-1-yl)Methyl]-2′-Deoxyuridines 5a–d

A nucleoside (0.5 mmol) was dissolved in dry pyridine (7 mL), then 4-monomethoxytrityl chloride (0.231 g, 0.75 mmol) was added. The reaction mixture was heated at 37 °C for 12 h. Then, the mixture was evaporated, dissolved in CHCl_3_, and extracted with 0.5 M sodium bicarbonate; the chloroform layer was washed with water. The organic layer was dried with Na_2_SO_4_. Then, the mixture was filtered, evaporated, and dissolved in dry pyridine (5 mL), and then acetic anhydride (0.077 g, 0.07 mL, 0.75 mmol) was added. The reaction mixture was stirred at 25 °C for 12 h, then evaporated, and the product was isolated by column chromatography in the chloroform/ethanol (60:1 *v*/*v*) eluting system with 2% DIPEA.

**3′-*O*-Acetyl-5′-*O*-monomethoxytrityl-5-undecyloxymethyl-2′-deoxyuridine (5a)** was prepared according to the general procedure from **1a** (0.206 g, 0.5 mmol). Yield: 0.330 g (91%).

^1^H NMR (300 MHz, DMSO-*d_6_*) δ 11.46 (s, 1H, 3-NH), 7.64 (s, 1H, 6-H), 7.44–7.20 (m, 12H, MMTr), 6.94–6.85 (m, 2H, MMTr), 6.18 (dd, *J* = 8.1, 6.2 Hz, 1H, 1′-H), 5.36–5.23 (m, 1H, 3′-H), 4.07 (q, *J* = 3.6 Hz, 1H, 4′-H), 3.88 (d, *J* = 11.5 Hz, 1H, 5-CH_2_), 3.75 (s, 3H, CH_3_O), 3.65 (d, *J* = 11.6 Hz, 1H, 5-CH_2_b), 3.33–3.25 (m, 2H, 5′-CH_2_), 3.15 (t, *J* = 6.6 Hz, 2H, α-CH_2_), 2.48–2.28 (m, 2H, 2-H′), 2.04 (s, 3H, Ac), 1.43–1.19 (m, 18H, (CH_2_)_8_, β-CH_2_), 0.88–0.79 (m, 3H, CH_3_).

**3′-*O*-Acetyl-5′-*O*-monomethoxytrityl-5-dodecyloxymethyl-2′-deoxyuridine (5b)** was prepared according to the general procedure from **1b** (0.213 g, 0.5 mmol). Yield: 0.370 g (89%).

^1^H NMR (300 MHz, DMSO-*d_6_*) δ 11.46 (s, 1H, 3-NH), 7.64 (s, 1H, 6-H), 7.46–7.19 (m, 12H, MMTr), 6.95–6.84 (m, 2H, MMTr), 6.18 (dd, *J* = 8.1, 6.2 Hz, 1H, 1′-H), 5.38–5.24 (m, 1H, 3′-H), 4.07 (q, *J* = 3.6 Hz, 1H, 4′-H), 3.88 (d, *J* = 11.5 Hz, 1H, 5-CH_2_a), 3.75 (s, 3H, CH_3_O), 3.65 (d, *J* = 11.6 Hz, 1H, 5-CH_2_b), 3.31–3.26 (m, 2H, 5′-CH_2_), 3.15 (t, *J* = 6.6 Hz, 2H, α-CH_2_), 2.47–2.27 (m, 2H, 2′-H), 2.05 (s, 3H, Ac), 1.37–1.16 (m, 20H, (CH_2_)_9_, β-CH_2_), 0.91–0.82 (m, 3H, CH_3_).

**3′-*O*-Acetyl-5′-*O*-monomethoxytrityl 5-[4-decyl-(1,2,3-triazol-1-yl)methyl]-2′-deoxyuridine (5c)** was prepared according to the general procedure from **1c** (0.224 g, 0.5 mmol). Yield: 0.343 g (90%).

^1^H NMR (300 MHz, DMSO-*d_6_*) δ 11.61 (s, 1H, 3-NH), 7.97 (s, 1H, H-Tri), 7.62 (s, 1H, 6-H), 7.45–7.17 (m, 12H, MMTr), 6.94–6.84 (m, 2H, MMTr), 6.18 (dd, *J* = 7.7, 6.3 Hz, 1H, 1′-H), 5.31 (dt, *J* = 6.6, 3.0 Hz, 1H, 3′-H), 4.80 (d, *J* = 14.3 Hz, 1H, 5-CH_2_a), 4.72–4.61 (m, 1H, 5-CH_2_b), 4.07 (q, *J* = 3.8 Hz, 1H, 4′-H), 3.74 (s, 3H, CH_3_O), 3.38 (dd, *J* = 10.4, 4.8 Hz, 1H, α-CH_2_a), 3.29–3.19 (m, 3H, 5′-CH_2_, α-CH_2_b), 2.54 (d, *J* = 7.7 Hz, 1H, 2′-Ha), 2.38 (ddd, *J* = 14.2, 6.4, 2.8 Hz, 1H, 2′-Hb), 2.05 (s, 3H, Ac), 1.64–1.43 (m, 2H, β-CH_2_), 1.38–1.14 (m, 14H, (CH_2_)_7_), 0.94–0.79 (m, 3H, CH_3_).

**3′-*O*-Acetyl-5′-*O*-monomethoxytrityl-5-[4-dodecyl-(1,2,3-triazol-1-yl)methyl]-2′-deoxyuridine (5d)** was prepared according to the general procedure from **1d** (0.238 g, 0.5 mmol). Yield: 0.356 g (90%).

^1^H NMR (300 MHz, DMSO-*d_6_*) δ 11.61 (s, 1H, 3-NH), 7.97 (s, 1H, H-Tri), 7.63 (s, 1H, 6-H), 7.43–7.17 (m, 12H, MMTr), 6.95–6.84 (m, 2H, MMTr), 6.18 (t, *J* = 7.0 Hz, 1H, 1′-H), 5.36–5.26 (m, 1H, 3′-H), 4.80 (d, *J* = 14.3 Hz, 1H, 5-CH_2_a), 4.67 (d, *J* = 14.3 Hz, 1H, 5-CH_2_b), 4.08 (q, *J* = 3.9 Hz, 1H, 4′-H), 3.73 (s, 3H, CH_3_O), 3.38 (dd, *J* = 10.5, 4.8 Hz, 1H, α-CH_2_a), 3.30–3.15 (m, 1H, 5′-CH_2_, α-CH_2_b), 2.54 (d, *J* = 7.3 Hz, 1H, 2′-Ha), 2.38 (ddd, *J* = 14.2, 6.3, 2.8 Hz, 1H, 2′-Hb), 2.05 (s, 3H, Ac), 1.61–1.43 (m, 2H, β-CH_2_), 1.35–1.18 (m, 18H, (CH_2_)_9_), 0.97–0.74 (m, 3H, CH_3_).

### 4.3. General Method for the Synthesis of 5′-O-Monomethoxytrityl-5-Alkyloxymethyl- and 5-[4-Alkyl-(1,2,3-Triazol-1-yl)Methyl]-4-Thio-2′-Deoxyuridines 6a–d

2-Chlorophenyl dichlorophosphate (0.216 g, 0.143 mL, 0.88 mmol) was added to a solution of protected nucleoside (0.40 mmol) and 1,2,4-triazole (0.165 g, 2.4 mmol) in anhydrous pyridine, cooled to 0 °C. The mixture was stirred for 24 h at room temperature and then evaporated. The residue was partitioned between chloroform and 0.5 M sodium bicarbonate; the chloroform layer was washed with water, dried over Na_2_SO_4_, evaporated, and dissolved in anhydrous dioxane (3 mL). Then, thioacetic acid (0.043 mL, 0.6 mmol) and pyridine (1 mL) were added to a solution. The mixture was stirred for 20 h at room temperature and then evaporated, dissolved in ethanol (3 mL), and then 3 mL of conc. aq. ammonia solution was added. The mixture was stirred for 12 h at room temperature and then evaporated. The product was isolated by column chromatography in the chloroform/ethanol (30:1 *v*/*v*) eluting system with 2% DIPEA. The target fractions were evaporated in a vacuum to give the expected compound yields as a yellow amorphous mass.

**5′-*O*-Monomethoxytrityl-5-undecyloxymethyl-4-thio-2′-deoxyuridine (6a)** was prepared according to the general procedure from **5a** (0.290 g, 0.4 mmol). Yield: 0.168 g (60%).

^1^H NMR (300 MHz, DMSO-*d_6_*) δ 12.78 (s, 1H, 3-NH), 7.54 (s, 1H, 6-H), 7.45–7.20 (m, 12H, MMTr), 6.98–6.79 (m, 2H, MMTr), 6.12 (t, *J* = 6.4 Hz, 1H, 1′-H), 5.36 (d, *J* = 4.6 Hz, 1H, 3′-OH), 4.24–4.12 (m, 2H, 5-CH_2_a, 3′-H), 4.02–3.89 (m, 2H, 5-CH_2_b, 4′-H), 3.75 (s, 3H, CH_3_O), 3.25–3.19 (m, 4H, 5′-CH_2_,α-CH_2_), 2.33–2.14 (m, 2H, 2′-H), 1.37–1.15 (m, 18H, (CH_2_)_8_, β-CH_2_), 0.90–0.80 (m, 3H, CH_3_).

**5′-*O*-Monomethoxytrityl-5-dodecyloxymethyl-4-thio-2′-deoxyuridine (6b)** was prepared according to the general procedure from **5b** (0.296 g, 0.4 mmol). Yield: 0.171 g (56%).

^1^H NMR (300 MHz, DMSO-*d_6_*) δ 12.77 (s, 1H, 3-NH), 7.54 (s, 1H, 6-H), 7.46–7.17 (m, 12H, MMTr), 6.94–6.83 (m, 2H, MMTr), 6.12 (t, *J* = 6.4 Hz, 1H, 1′-H), 5.34 (d, *J* = 4.6 Hz, 1H, 3′-OH), 4.21–4.15 (m, 2H, 5-CH_2_a, 3′-H), 4.02–3.89 (m, 2H, 5-CH_2_b, 4′-H), 3.75 (s, 3H, CH_3_O), 3.24–3.18 (m, 4H, 5′-CH_2_,α-CH_2_), 2.32–2.14 (m, 2H, 2′-H), 1.28–1.18 (m, 20H, (CH_2_)_9_, β-CH_2_), 0.92–0.79 (m, 3H, CH_3_).

**5′-*O*-Monomethoxytrityl-5-[4-decyl-(1,2,3-triazol-1-yl)methyl]-4-thio-2′-deoxyuridine (6c)** was prepared according to the general procedure from **5c** (0.305 g, 0.4 mmol). Yield: 0.162 g (55%).

^1^H NMR (300 MHz, DMSO-*d_6_*) δ 12.93 (s, 1H, 3-NH), 7.91 (s, 1H, H-Tri), 7.66 (s, 1H, 6-H), 7.39 (s, 12H, MMTr), 6.92–6.82 (m, 2H, MMTr), 6.11 (t, *J* = 6.2 Hz, 1H, 1′-H), 5.36 (d, *J* = 4.6 Hz, 1H, 3′-OH), 5.09 (d, *J* = 14.2 Hz, 1H, 5-CH_2_a), 4.83 (d, *J* = 14.2 Hz, 1H, 5-CH_2_b), 4.36–4.19 (m, 1H, 3′-H), 3.93 (td, *J* = 4.8, 3.2 Hz, 1H, 4′-H), 3.75 (s, 3H, CH_3_O), 3.36–3.29 (m, 2H, 5′-CH_2_), 3.29–3.24 (m, 1H, α-CH_2_a), 3.18 (dd, *J* = 10.5, 3.2 Hz, 1H, α-CH_2_b), 2.39–2.21 (m, 2H, 2′-H), 1.60–1.42 (m, 2H, β-CH_2_), 1.28–1.21 (m, 16H, (CH_2_)_8_), 0.92–0.77 (m, 3H, CH_3_).

**5′-*O*-Monomethoxytrityl-5-[4-dodecyl-(1,2,3-triazol-1-yl)methyl]-4-thio-2′-deoxyuridine (6d)** was prepared according to the general procedure from **5d** (0.316 g, 0.4 mmol). Yield: 0.177 g (58%).

^1^H NMR (300 MHz, DMSO-*d_6_*) δ 7.91 (s, 1H, H-Tri), 7.65 (s, 1H, 6-H), 7.44–7.17 (m, 12H, MMTr), 6.93–6.81 (m, 2H, MMTr), 6.11 (t, *J* = 6.2 Hz, 1H, 1′-H), 5.36 (d, *J* = 4.8 Hz, 1H, 3′-OH), 5.08 (d, *J* = 14.2 Hz, 1H, 5-CH_2_a), 4.82 (d, *J* = 14.2 Hz, 1H, 5-CH_2_b), 4.34–4.22 (m, 1H, 3′-H), 3.95–3.88 (m, 1H, 4′-H), 3.73 (s, 3H, CH_3_O), 3.30–3.27 (m, 2H, 5′-CH_2_), 3.27–3.21 (m, 1H, α-CH_2_a), 3.18 (dd, *J* = 10.6, 3.2 Hz, 1H, α-CH_2_b), 2.32–2.23 (m, 2H, 2′-H), 1.57–1.46 (m, 2H, β-CH_2_), 1.25–1.21 (m, 18H, (CH_2_)_9_), 0.94–0.80 (m, 3H, CH_3_).

### 4.4. General Method for the Synthesis of 5-Alkyloxymethyl-and 5-[4-Alkyl-(1,2,3-Triazol-1-yl)Methyl]-4-Thio-2′-Deoxyuridines 3a–d

A nucleoside (0.10 mmol) was dissolved in an aqueous (80%) solution of acetic acid (7 mL); then, the mixture was stirred for 12 h at room temperature and then evaporated. The product was isolated by column chromatography in the chloroform/ethanol (9:1 *v*/*v*) eluting system.

**5-Undecyloxymethyl-4-thio-2′-deoxyuridine (3a)** was prepared according to the general procedure from **6a** (0.07 g, 0.10 mmol). Yield: 0.041 g (96%). UV: λ_max_ 334 nm. ^1^H NMR (300 MHz, DMSO-*d_6_*) δ 12.72 (s, 1H, 3-NH), 7.83 (t, *J* = 0.9 Hz, 1H, 6-H), 6.12 (t, *J* = 6.6 Hz, 1H, 1′-H), 5.26 (d, *J* = 4.1 Hz, 1H, 3′-OH), 4.95 (t, *J* = 5.0 Hz, 1H, 5′-OH), 4.28 (d, *J* = 1.0 Hz, 2H, 5-CH_2_), 4.23 (dt, *J* = 6.4, 3.4 Hz, 1H, 3′-H), 3.84 (q, *J* = 3.8 Hz, 1H, 4′-H), 3.62–3.51 (m, 2H, 5′-CH_2_), 3.44 (t, *J* = 6.5 Hz, 2H, α-CH_2_), 2.27–2.00 (m, 2H, 2′-H), 1.60–1.44 (m, 2H, β-CH_2_), 1.33–1.21 (m, 16H, (CH_2_)_8_), 0.86 (t, *J* = 6.8 Hz, 3H, CH_3_). ^13^C NMR (75 MHz, DMSO-*d_6_*) δ 188.69 (C-4), 147.46 (C-2), 134.23 (C-6), 118.83 (C-5), 87.81 (C-4′), 85.11 (C-1′), 70.35 (C-3′), 70.13 (α-CH_2_), 67.20 (5-CH_2_), 61.23 (C-5′), 39.99 (C-2′), 31.26, 29.11, 29.00, 28.83, 28.68, 25.62, 22.05 ((CH_2_)_9_), 13.89 (CH_3_). HRMS (ESI) of C_21_H_36_N_2_O_5_S *m*/*z:* calcd [M − H]^−^ 427.2261, found: 427.2280.

**5-Dodecyloxymethyl-4-thio-2′-deoxyuridine (3b)** was prepared according to the general procedure from **6b** (0.071 g, 0.10 mmol). Yield: 0.042 g (95%). UV: λ_max_ 334 nm. ^1^H NMR (300 MHz, DMSO-*d_6_*) δ 12.72 (s, 1H, 3-NH), 7.83 (t, *J* = 0.9 Hz, 1H, 6-H), 6.12 (t, *J* = 6.6 Hz, 1H, 1′-H), 5.26 (d, *J* = 4.2 Hz, 1H, 3′-OH), 4.96 (t, *J* = 5.1 Hz, 1H, 5′-OH), 4.28 (d, *J* = 1.0 Hz, 2H, 5-CH_2_), 4.23 (dt, *J* = 6.9, 3.4 Hz, 1H, 3′-H), 3.84 (q, *J* = 3.8 Hz, 1H, 4′-H), 3.61–3.53 (m, 2H, 5′-CH_2_), 3.44 (t, *J* = 6.5 Hz, 2H, α-CH_2_), 2.26–2.04 (m, 2H, 2′-H), 1.59–1.44 (m, 2H, β-CH_2_), 1.36–1.20 (m, 18H, (CH_2_)_9_), 0.86 (t, *J* = 6.8 Hz, 3H, CH_3_). ^13^C NMR (75 MHz, DMSO-*d_6_*) δ 188.70 (C-4), 147.47 (C-2), 134.25 (C-6), 118.83 (C-5), 87.81 (C-4′), 85.12 (C-1′), 70.35 (C-3′), 70.13 (α-CH_2_), 67.20 (5-CH_2_), 61.23 (C-5′), 39.99 (C-2′), 31.26, 29.33–28.42, 25.62, 22.05 ((CH_2_)_10_), 13.90 (CH_3_). HRMS (ESI) of C_22_H_38_N_2_O_5_S *m*/*z*: calcd [M − H]^−^ 441.2418, found: 441.2414.

**5-[4-Decyl-(1,2,3-triazol-1-yl)methyl]-4-thio-2′-deoxyuridine (3c)** was prepared according to the general procedure from **6c** (0.074 g, 0.10 mmol). Yield: 0.044 g (95%). UV: λ_max_ 332 nm. ^1^H NMR (300 MHz, DMSO-*d_6_*) δ 12.88 (s, 1H, 3-NH), 8.05 (s, 1H, H-Tri), 7.79 (t, *J* = 0.7 Hz, 1H, 6-H), 6.07 (t, *J* = 6.4 Hz, 1H, 1′-H), 5.41–5.31 (m, 2H, 5-CH_2_), 4.23 (dt, *J* = 5.9, 3.7 Hz, 1H, 3′-H), 3.83 (q, *J* = 3.9 Hz, 1H, 4′-H), 3.62–3.48 (m, 2H, 5′-CH_2_), 2.57 (t, *J* = 7.6 Hz, 2H, α-CH_2_), 2.29–2.09 (m, 2H, 2′-H), 1.68–1.45 (m, 2H, β-CH_2_), 1.34–1.20 (m, 14H, (CH_2_)_7_), 0.86 (t, *J* = 6.8 Hz, 3H, CH_3_). ^13^C NMR (75 MHz, DMSO-*d_6_*) δ 189.02 (C-4), 147.37 (C-2), 146.39 (C-C_10_H_21_), 137.55 (C-6), 121.98 (CH(Tri)), 115.82 (C-5), 87.91 (C-4′), 85.48 (C-1′), 69.92 (C-3′), 60.93 (C-5′), 48.67 (5-CH_2_), 40.05 (C-2′), 31.25, 28.94, 28.69, 24.97, 22.05 (Tri(CH_2_)_9_), 13.89 (CH_3_). HRMS (ESI) of C_22_H_35_N_5_O_4_S *m*/*z*: calcd [M − H]^−^ 464.2326, found: 464.2358.

**5-[4-Dodecyl-(1,2,3-triazol-1-yl)methyl]-4-thio-2′-deoxyuridine (3d)** was prepared according to the general procedure from **6d** (0.076 g, 0.10 mmol). Yield: 0.046 g (95%). UV: λ_max_ 332 nm. ^1^H NMR (300 MHz, DMSO-*d_6_*) δ 12.87 (s, 1H, 3-NH), 8.05 (s, 1H, H-Tri), 7.79 (t, *J* = 0.7 Hz, 1H, 6-H), 6.10–6.04 (m, 1H, 1′-H), 5.42–5.30 (m, *J* = 2.7 Hz, 2H, 5-CH_2_), 4.29–4.18 (m, 1H, 3′-H), 3.83 (q, *J* = 3.9 Hz, 1H, 4′-H) 3.57–3.52 (m, 2H, 5′-CH_2_), 2.57 (t, *J* = 7.6 Hz, 2H, α-CH_2_), 2.29–2.08 (m, 2H, 2′-H), 1.66–1.47 (m, 2H, β-CH_2_), 1.34–1.14 (m, 18H, (CH_2_)_9_), 0.86 (t, *J* = 6.8 Hz, 3H, CH_3_). ^13^C NMR (75 MHz, DMSO-*d_6_*) δ 189.02 (C-4), 147.34 (C-2), 146.39 (C-C_12_H_25_), 137.54 (C-6), 121.95 (CH(Tri)), 115.83 (C-5), 87.91 (C-4′), 85.48 (C-1′), 69.92 (C-3′), 60.93 (C-5′), 48.65 (5-CH_2_), 37.33 (C-2′), 31.24, 28.94, 28.80, 28.48, 24.97, 22.04 (Tri(CH_2_)_11_), 13.89 (CH_3_). HRMS (ESI) of C_24_H_39_N_5_O_4_S *m*/*z*: calcd [M − H]^−^ 492.2639, found: 492.2685.

### 4.5. General Method for the Synthesis of 3′-O-(8-Hydroxy-3,6-Dioxaoct-1-Yloxy)Carbonyl-5-Alkyloxymethyl- and 5-[4-Alkyl-(1,2,3-Triazol-1-yl)Methyl]-4-Thio-2′-Deoxyuridines 4a–d

A nucleoside (0.2 mmol) was dissolved in dry dimethylformamide (2 mL), and CDI was added (0.129 g, 0.8 mmol). The mixture was heated at 37 °C for 24 h. Then, the anhydrous triethylene glycol (0.6 g, 0.55 mL, 4 mmol) and dioxane (1 mL) were added. The mixture was heated at 37 °C for 24 h and then evaporated to obtain crude oil with constant volume. The product was extracted in the chloroform–water system, the organic layer was evaporated, and then dissolved in an aqueous (80%) solution of acetic acid (5 mL). The mixture was stirred for 12 h at room temperature and then evaporated. The product was isolated by column chromatography in the chloroform/ethyl acetate/ethanol (10:10:1 *v*/*v*) eluting system.

**3′-*O*-(8-Hydroxy-3,6-dioxaoct-1-yloxy)carbonyl-5-undecyloxymethyl-4-thio-2′-deoxyuridine (4a)** was prepared according to the general procedure from **6a** (0.140 g, 0.20 mmol). Yield: 0.075 g (62%). UV: λ_max_ 334 nm. ^1^H NMR (300 MHz, Chloroform-*d*) δ 7.70 (t, *J* = 1.4 Hz, 1H, 6-H), 6.19 (t, *J* = 7.1 Hz, 1H, 1′-H), 5.31 (ddd, *J* = 4.5, 4.1, 2.0 Hz, 1H, 3′-H), 4.47–4.38 (m, 2H, 5-CH_2_), 4.38–4.30 (m, 2H, O-C(O)-OCH_2_(TEG)), 4.25 (q, *J* = 2.6 Hz, 1H, 4′-H), 3.99–3.85 (m, 2H, 5′-CH_2_), 3.81–3.60 (m, 10H, 5×CH_2_ (TEG)), 3.56 (t, *J* = 6.7 Hz, 2H, α-CH_2_), 2.61–2.50 (m, 2H, 2′-H), 1.68–1.56 (m, 2H, β-CH_2_), 1.40–1.23 (m, 16H, (CH_2_)_8_), 0.90 (t, *J* = 6.8 Hz, 3H, CH_3_). ^13^C NMR (75 MHz, Chloroform-*d*) δ 187.98 (C-4), 154.40 (O-C(O)-O), 147.57 (C-2), 133.77 (C-6), 120.74 (C-5), 87.77 (C-1′), 85.45 (C-4′), 78.43 (C-3′), 72.48 (TEG), 71.56 (α-CH_2_), 70.66 (TEG), 70.33 (TEG), 68.80 (TEG), 67.53 (5-CH_2_), 67.34 (TEG), 62.57 (C-5′), 61.72 (TEG), 37.37 (C-2′), 31.90, 29.76, 29.14, 26.11, 22.67 ((CH_2_)_9_), 14.09 (CH_3_). HRMS (ESI) of C_28_H_48_N_2_O_10_S *m*/*z*: calcd [M − H]^−^ 603.2946, found: 603.2943.

**3′-*O*-(8-Hydroxy-3,6-dioxaoct-1-yloxy)carbonyl-5-dodecyloxymethyl-4-thio-2′-deoxyuridine (4b)** was prepared according to the general procedure from **6b** (0.142 g, 0.20 mmol). Yield: 0.067 g (54%). UV: λ_max_ 334 nm. ^1^H NMR (300 MHz, DMSO-*d_6_*) δ 12.77 (s, 1H, 3-NH), 7.86 (t, *J* = 1.0 Hz, 1H, 6-H), 6.13 (dd, *J* = 8.2, 5.9 Hz, 1H, 1′-H), 5.24–5.17 (m, 1H, 3′-H), 5.17–5.10 (m, 1H, 5′-OH), 4.54 (br. s, 1H, CH_2_OH), 4.28 (d, *J* = 1.0 Hz, 2H, 5-CH_2_), 4.26–4.20 (m, 2H, C(O)-OCH_2_(TEG)), 4.14 (td, *J* = 3.5, 1.7 Hz, 1H, 4′-H), 3.71– 3.39 (m, 14H, 5′-CH_2_, 5×CH_2_ (TEG), α-CH_2_), 2.47–2.28 (m, 2H, 2′-H), 1.60–1.46 (m, 2H, β-CH_2_), 1.35–1.20 (m, 18H, (CH_2_)_9_), 0.86 (t, *J* = 6.8 Hz, 3H, CH_3_). ^13^C NMR (75 MHz, DMSO-*d_6_*) δ 188.86 (C-4), 153.79 (O-C(O)-O), 147.43 (C-2), 134.00 (C-6), 119.04 (C-5), 85.05 (C-1′), 84.94 (C-4′), 78.44 (C-3′), 72.32 (TEG), 70.16 (TEG), 69.74, 69.71 (TEG, α-CH_2_), 68.06 (TEG), 67.17 (5-CH_2_), 67.08 (TEG), 61.20 (TEG), 60.18 (C-5′), 37.15 (C-2′), 31.25, 29.28, 28.78, 28.67, 25.60, 22.04 ((CH_2_)_10_), 13.89 (CH_3_). HRMS (ESI) of C_29_H_50_N_2_O_10_S *m*/*z*: calcd [M + H]^+^ 619.3259, found: 619.3264.

**3′-*O*-(8-Hydroxy-3,6-dioxaoct-1-yloxy)carbonyl-5-[4-decyl-(1,2,3-triazol-1-yl)methyl]-4-thio-2′-deoxyuridine (4c)** was prepared according to the general procedure from **6c** (0.147 g, 0.20 mmol). Yield: 0.064 g (50%). UV: λ_max_ 332 nm. ^1^H NMR (300 MHz, Chloroform-*d*) δ 8.19 (s, 1H, H-Tri), 7.59 (t, *J* = 0.7 Hz, 1H, 6-H), 6.23 (dd, *J* = 7.7, 5.9 Hz, 1H, 1′-H), 5.59–5.37 (m, 2H, 5-CH_2_), 5.26 (dt, *J* = 6.2, 1.8 Hz, 1H, 3′-H), 4.35–4.29 (m, 2H, C(O)-OCH_2_(TEG)), 4.29–4.24 (m, 1H, 4′-H), 3.95–3.81 (m, 2H, 5′-CH_2_), 3.79–3.59 (m, 10H, 5×CH_2_ (TEG)), 2.74–2.64 (m, 2H, α-CH_2_), 2.64–2.54 (m, 1H, 2′-Ha), 2.36 (ddd, *J* = 14.1, 7.8, 6.1 Hz, 1H, 2′-Hb), 1.73–1.57 (m, 2H, β-CH_2_), 1.35–1.22 (m, 14H, (CH_2_)_7_), 0.90 (t, *J* = 6.8 Hz, 3H, CH_3_). ^13^C NMR (75 MHz, Chloroform-*d*) δ 188.59 (C-4), 154.39 (O-C(O)-O), 148.58, 147.48 (C-2, C-C_10_H_21_), 137.00 (C-6), 122.70 (CH(Tri)), 117.45 (C-5), 86.99 (C-1′), 86.19 (C-4′), 78.98 (C-3′), 72.64 (TEG), 70.77 (TEG), 70.44 (TEG), 68.88 (TEG), 67.39 (TEG), 62.16 (C-5′), 61.74 (TEG), 49.00 (5-CH_2_), 38.85 (C-2′), 32.00, 29.68, 29.53, 29.32, 25.69, 22.77 ((CH_2_)_9_), 14.21 (CH_3_). HRMS (ESI) of C_29_H_47_N_5_O_9_S *m*/*z*: calcd [M + H]^+^ 642.3167, found: 642.3120.

**3′-*O*-(8-Hydroxy-3,6-dioxaoct-1-yloxy)carbonyl-5-[4-dodecyl-(1,2,3-triazol-1-yl)methyl]-4-thio-2′-deoxyuridine (4d)** was prepared according to the general procedure from **6d** (0.153 g, 0.20 mmol). Yield: 0.078 g (58%). UV: λ_max_ 332 nm. ^1^H NMR (300 MHz, Chloroform-*d*) δ 10.45 (s, 1H, 3-NH), 8.18 (s, 1H, H-Tri), 7.58 (t, *J* = 0.7 Hz, 1H, 6-H), 6.23 (dd, *J* = 7.7, 5.9 Hz, 1H, 1′-H), 5.59–5.37 (m, 2H, 5-CH_2_), 5.26 (dt, *J* = 6.2, 1.8 Hz, 1H, 3′-H), 4.42–4.16 (m, 3H, CH_2_(TEG)), 4′-H), 3.95–3.81 (m, 2H, 5′-CH_2_), 3.81–3.56 (m, 10H, 5×CH_2_ (TEG)), 2.73–2.55 (m, 3H, α-CH_2_, 2′-Ha), 2.37 (ddd, *J* = 14.1, 7.8, 6.1 Hz, 1H, 2′-Hb), 1.73–1.55 (m, 2H, β-CH_2_), 1.38–1.12 (m, 18H, (CH_2_)_9_), 0.86 (t, *J* = 6.8 Hz, 3H, CH_3_). ^13^C NMR (75 MHz, Chloroform-*d*) δ 188.39 (C-4), 154.28 (O-C(O)-O), 148.52, 147.32 (C-2, C-C_12_H_25_), 136.86 (C-6), 122.58 (CH(Tri)), 117.31 (C-5), 86.95 (C-1′), 86.10 (C-4′), 78.86 (C-3′), 72.53 (TEG), 70.66 (TEG), 70.34 (TEG), 68.77 (TEG), 67.29 (TEG), 62.08 (C-5′), 61.65 (TEG), 48.88 (5-CH_2_), 38.77 (C-2′), 31.91, 29.89, 29.12, 25.59, 22.67 ((CH_2_)_10_), 14.10 (CH_3_). HRMS (ESI) of C_31_H_51_N_5_O_9_S m/z: calcd [M + H]^+^ 670.3480, found: 670.3423.

### 4.6. General Method for the Synthesis of 5-Alkyloxymethyl-and 5-[4-Alkyl-(1,2,3-Triazol-1-yl)Methyl]-4-Thiouridines 3e–h

A nucleoside (0.5 mmol) prepared as described in [48] was dissolved in dry dichloroethane (20 mL), then dimethylformamide (2 mL), and SOCl_2_ was added (0.129 g, 0.8 mmol, 0.079 mL). The reaction mixture was refluxed for 6 h. Then, the mixture was evaporated and co-evaporated with toluene two times and dissolved in dry dioxane (5 mL). Then, thioacetic acid (0.054 mL, 0.75 mmol) and pyridine (1 mL) were added to a solution. The mixture was stirred for 20 h at room temperature and then evaporated, dissolved in ethanol (5 mL), and then 5 mL of conc. aq. ammonia solution was added. The mixture was stirred for 12 h at room temperature and then evaporated. The product was isolated by column chromatography in the chloroform/ethanol (20:1 *v*/*v*) eluting system. The target fractions were evaporated in a vacuum to give the expected compound yields as a yellow powder.

**5-Undecyloxymethyl-4-thiouridine (3e)** was prepared according to the general procedure from **7e** (0.277 g, 0.50 mmol). Yield: 0.119 g (54%). UV: λ_max_ 330 nm. ^1^H NMR (300 MHz, DMSO-*d_6_*) δ 12.74 (s, 1H, 3-NH), 7.94–7.88 (m, 1H, 6-H), 5.79 (d, *J* = 4.9 Hz, 1H, 1′-H), 5.44 (br. s, 1H, 2′-OH), 5.16–4.96 (m, 2H, 3′-OH, 5′-OH), 4.33–4.21 (m, 2H, 5-CH_2_), 4.09–4.02 (m, 1H, 2′-H), 4.00–3.94 (m, 1H, 3′-H), 3.93–3.85 (m, 1H, 4′-H), 3.71–3.50 (m, 2H, 5′-CH_2_), 3.43 (t, *J* = 6.6 Hz, 2H, α-CH_2_), 1.58–1.45 (m, 2H, β-CH_2_), 1.31–1.23 (m, 16H, (CH_2_)_8_), 0.86 (t, *J* = 6.8 Hz, 3H, CH_3_). ^13^C NMR (75 MHz, DMSO-*d_6_*) δ 188.95 (C-4), 147.78 (C-2), 134.63 (C-6), 118.90 (C-5), 88.43 (C-1′), 85.07 (C-4′), 73.97 (C-2′), 70.16 (C-3′), 69.86 (α-CH_2_), 67.21 (5-CH_2_), 60.77 (C-5′), 31.28, 29.21, 28.77, 28.69, 25.63, 22.46, 22.07, ((CH_2_)_9_), 13.92 (CH_3_). HRMS (ESI) of C_21_H_36_N_2_O_6_S *m*/*z*: calcd [M − H]^−^ 443.2210, found: 443.2215.

**5-Dodecyloxymethyl-4-thiouridine (3f)** was prepared according to the general procedure from **7f** (0.284 g, 0.50 mmol). Yield: 0.114 g (50%). UV: λ_max_ 330 nm.^1^H NMR (300 MHz, DMSO-*d_6_*) δ 12.73 (s, 1H, 3-NH), 8.00–7.79 (m, 1H, 6-H), 5.79 (d, *J* = 4.9 Hz, 1H, 1′-H), 5.44 (d, *J* = 5.4 Hz, 1H, 2′-OH), 5.09 (d, *J* = 5.2 Hz, 1H, 3′-OH), 5.05 (t, *J* = 5.0 Hz, 1H, 5′-OH), 4.33–4.22 (m, 2H, 5-CH_2_), 4.06 (dt, *J* = 5.1, 5.0 Hz, 1H, 2′-H), 3.97 (td, *J* = 5.1, 4.4 Hz, 1H, 3′-H), 3.90 (dt, *J* = 4.4, 3.5 Hz, 1H, 4′-H), 3.61 (qdd, *J* = 12.0, 4.9, 3.2 Hz, 2H, 5′-CH_2_), 3.43 (t, *J* = 6.6 Hz, 2H, α-CH_2_), 1.59–1.45 (m, 2H, β-CH_2_), 1.39–1.15 (m, 18H, (CH_2_)_9_), 0.86 (t, *J* = 6.8 Hz, 3H, CH_3_). ^13^C NMR (75 MHz, DMSO-*d_6_*) δ 188.94 (C-4), 147.77 (C-2), 134.62 (C-6), 118.89 (C-5), 88.43 (C-1′), 85.07 (C-4′), 73.97 (C-2′), 70.16 (C-3′), 69.86 (α-CH_2_), 67.21 (5-CH_2_), 60.77 (C-5′), 31.28, 29.08, 29.02, 28.86, 28.70, 25.63, 22.08 ((CH_2_)_10_), 13.92 (CH_3_). HRMS (ESI) of C_22_H_38_N_2_O_6_S m/z: calcd [M − H]^−^ 457.2367, found: 457.2363.

**5-[4-Decyl-(1,2,3-triazol-1-yl)methyl]-4-thiouridine (3g)** was prepared according to the general procedure from **7g** (0.295 g, 0.50 mmol). Yield: 0.132 g (55%). UV: λ_max_ 328 nm.^1^H NMR (300 MHz, DMSO-*d_6_*) δ 12.89 (s, 1H, 3-NH), 8.21 (s, 1H, H-Tri), 7.79 (s, 1H, 6-H), 5.73 (d, *J* = 4.1 Hz, 1H, 1′-H), 5.39–5.28 (m, 2H, 5-CH_2_), 4.07 (dd, *J* = 5.0, 4.1 Hz, 1H, 2′-H), 3.98 (dd, *J* = 5.5, 5.0 Hz, 1H, 3′-H), 3.89 (dt, *J* = 5.5, 3.2 Hz, 1H, 4′-H), 3.69 (dd, *J* = 12.2, 3.2 Hz, 1H, 5′-CH_2_a), 3.56 (dd, *J* = 12.2, 3.4 Hz, 1H, 5′-CH_2_b), 2.57 (t, *J* = 7.6 Hz, 2H, α-CH_2_), 1.62–1.49 (m, 2H, β-CH_2_), 1.31–1.21 (m, 14H, (CH_2_)_7_), 0.86 (t, *J* = 6.8, 3H, CH_3_). ^13^C NMR (75 MHz, DMSO-*d_6_*) δ 189.20 (C-4), 147.60 (C-2), 146.38 (C-C_10_H_21_), 137.92 (C-6), 121.99 (CH-Tri), 115.84 (C-5), 89.08 (C-1′), 84.84 (C-4′), 73.99 (C-2′), 69.19 (C-3′), 60.33 (C-5′), 48.72 (5-CH_2_), 31.27, 28.97, 28.81, 28.55, 25.00, 22.07 ((CH_2_)_9_), 13.91 (CH_3_). HRMS (ESI) of C_22_H_35_N_5_O_5_S m/z: calcd [M + H]^+^: 482.2432, found: 482.2417.

**5-[4-Dodecyl-(1,2,3-triazol-1-yl)methyl]-4-thiouridine (3h)** was prepared according to the general procedure from **7h** (0.309 g, 0.50 mmol). Yield: 0.142 g (56%). UV: λ_max_ 328 nm. ^1^H NMR (300 MHz, DMSO-*d_6_*) δ 12.89 (s, 1H, 3-NH), 8.21 (s, 1H, H-Tri), 7.79 (s, 1H, 6-H), 5.73 (d, *J* = 4.5 Hz, 1H, 1′-H), 5.49–5.18 (m, 2H, 5-CH_2_), 4.07 (t, *J* = 4.5 Hz, 1H, 2′-H), 3.98 (t, *J* = 5.0 Hz, 1H, 3′-H), 3.92–3.86 (m, 1H, 4′-H), 3.69 (dd, *J* = 12.2, 3.2 Hz, 1H, 5′-CH_2_a), 3.56 (dd, *J* = 12.3, 3.3 Hz, 1H, 5′-CH_2_b), 2.58 (t, *J* = 6.6 Hz, 2H, α-CH_2_), 1.63–1.46 (m, 2H, β-CH_2_), 1.28–1.20 (m, 18H, (CH_2_)_9_), 0.86 (t, *J* = 6.8, 3H, CH_3_). ^13^C NMR (75 MHz, DMSO-*d_6_*) δ 189.17 (C-4), 147.57 (C-2), 146.35 (C-C_12_H_25_), 137.91 (C-6), 121.98 (CH-Tri), 115.81 (C-5), 89.06 (C-1′), 84.82 (C-4′), 73.97 (C-2′), 69.17 (C-3′), 60.31 (C-5′), 48.71 (5-CH_2_), 31.25, 28.98, 28.86, 28.26, 24.98, 22.05 ((CH_2_)_11_), 13.90 (CH_3_). HRMS (ESI) of C_24_H_39_N_5_O_5_S m/z: calcd [M + H]^+^: 510.2745, found: 510.2734.

### 4.7. Bacterial Strains and In Vitro Study of the Antibacterial Effect

The following test strains were used: Gram-positive bacteria *Micrococcus luteus* NCTC 8340, *Staphylococcus aureus* FDA 209P and INA 00761 (MRSA); mycobacteria *Mycobacterium smegmatis* mc^2^ 155, and *M. smegmatis* VKPM Ac-1339, all from the collection of the Gause Institute of New Antibiotics.

Test strains were incubated in modified Gause’s nutrient medium liquid № 2. The level of infection with test cultures was 10^6^ cells/mL. A compound being tested was dissolved in 30% aq. methanol. Ten volume percent of the tested compound solution was added to the nutrient medium. Samples without the addition of substances, antibiotics in medical use (amikacin, ciprofloxacin, isoniazid, rifampicin, oxacillin, and vancomycin), and samples of medium supplemented with a mixture of solvent served as control of the test culture growth. All strains were incubated at 37°C.

### 4.8. Mycobacterial Strains and Susceptibility Testing of Mycobacteria

The following strains from the laboratory collection were used: *M. smegmatis* mc^2^ 155, *M. fortuitum* ATCC 6841. Clinical isolates of *M. abscessuss* (N24-001, N24-009, N24-020, N24-021), *M. avium* (N28-003, N26-008), and *M. intracellulare* (N24-016, N24-017, N24-003) were obtained from the confirmed infection cases upon the study of nontuberculous mycobacteria diversity in Russia [70]. The MIC values for *Mycobacterium* species were determined by the broth microdilution method using the 96-well plates following the CLSI guidelines [71]. The cation-adjusted Mueller–Hinton broth (HiMedia Laboratories Pvt. Limited, Maharashtra, India, M391) with 5% OADC (HiMedia Laboratories Pvt. Limited, FD018) addition was used. All compounds were dissolved in DMSO to an 8 mg/L concentration, used as the starting solution for the 2× dilutions. Each well contained 200 μL of the dissolved bacterial cells and 2 μL of the DMSO solution of the compounds. The growth of the strains and isolates in wells were recorded by the addition of resazurin (Sigma-Aldrich, R7017) and visual inspection after 1–2 days for fast-growing species and 2–6 days for slow-growing species [72].

The susceptibility of mycobacterial strains and isolates to clarithromycin, rifampicin, ethambutol, and amikacin (all from Sigma-Aldrich), used for the treatment of nontuberculous mycobacterial infections, was also tested in the same conditions. The only exception was the use of water was used for dissolving amikacin. The MIC values obtained fit perfectly with the previously reported ranges [61].

## 5. Conclusions

In summary, we have developed a convenient method for the preparation of 4-thioanalogs of 5-substituted (5-alkyloxymethyl and 5-alkyltriazolylmethyl) derivatives of 2′-deoxyuridine and uridine. We have demonstrated that the substitution of an oxygen atom in the C4 position of modified nucleosides by a sulfur one leads to a significant increase in their antibacterial activity compared to oxygen-containing analogs. Obtained compounds showed noticeable in vitro activity against *M. smegmatis*, *S. aureus*, and *Mic. luteus* with the MIC range comparable to some clinically used antibiotics. Of particular interest is the inhibitory effect of newly synthesized compounds toward a set of mycobacteria that are characterized by intrinsic resistance to a wide range of antibacterial drugs. Two compounds (**3c**, **3e**) are active toward both laboratory strains and clinical isolates—the causative agents of mycobacterial diseases, including slow-growing *M. avium* and *M. intracellulare*, and rapidly growing *M. abscessus* and *M. fortuitum*. The obtained nucleoside derivatives can serve as promising lead compounds for the development of new antibacterial (primarily antimycobacterial) agents.

## Figures and Tables

**Table 1:** Water solubility of the synthesized compounds.

Compound	Lipophilic Substituent	Sugar Substituent	Water Solubility (mg/mL)
**3a**	-CH_2_OC_11_H_23_	-	<0.013
**3b**	-CH_2_OC_12_H_25_	-	0.013
**3c**	-CH_2_TriC_10_H_21_	-	0.014
**3d**	-CH_2_TriC_12_H_25_	-	0.005
**3e**	-CH_2_OC_11_H_23_	-	0.2
**3f**	-CH_2_OC_12_H_25_	-	0.013
**3g**	-CH_2_TriC_10_H_21_	-	0.01
**3h**	-CH_2_TriC_12_H_25_	-	<0.01
**4a**	-CH_2_OC_11_H_23_	3′-O-C(O)-OTEG	0.1
**4b**	-CH_2_OC_12_H_25_	3′-O-C(O)-OTEG	0.028
**4c**	-CH_2_TriC_10_H_21_	3′-O-C(O)-OTEG	0.6
**4d**	-CH_2_TriC_12_H_25_	3′-O-C(O)-OTEG	<0.1

**Table 2:** Bacterial growth minimal inhibitory concentration (MIC, mg/L) of obtained compounds.

Compound	MIC [mg/L]				
	*S. aureus* FDA 209P (MSSA)	*S. aureus* INA 00761 (MRSA)	*Mic. luteus* NCTC 8340	*M. smegmatis* VKPM Ac-1339	*M. smegmatis* mc^2^ 155
**4a**	5	5	5	5	5
**4b**	21	21	21	21	21
**2a**	n/a	n/a	n/a	24	n/a
**2b**	50	50	50	25	25
**2c**	78	n/a	n/a	n/a	n/a
**2d, 3e** **–3h, 4c, 4d**	n/a	n/a	n/a	n/a	n/a
**Amikacin**	2	30	0.5	30	-
**Ciprofloxacin**	4	4	-	4	-
**Isoniazid**	-	-	-	0.25	4
**Rifampicin**	-	-	-	8	4
**Oxacillin**	1	32	-	-	-

n/a—compound was not active at concentrations < 100 mg/L.

**Table 3:** Minimal mycobacterial growth inhibitory concentration (mg/L) of obtained compounds.

Compound	MIC [mg/L]			
	*M. abscessus*	*M. fortuitum*	*M. intracellulare*	*M. avium*
**3a**	80	>80	>80	>80
**3b**	40	>80	>80	>80
**3c**	40	20	10	20
**3d**	>80	>80	>80	>80
**3e**	20	20	10	20
**3f**	>80	>80	10	40
**3g**	>80	80	40	40
**3h**	>80	>80	>80	>80
**4a**	>80	>80	10	40
**4b**	>80	>80	20	40
**4c**	>80	>80	20	20
**4d**	>80	>80	40	80
**1b**	>80	>80	80	>80
**1e**	>80	>80	80	80
**1a,c,d,f–h**	>80	>80	>80	>80
**Clarithromycin**	1.25	20	>80	>80
**Ethambutol**	80	10	80	40
**Rifampicin**	>80	20	1.25	1.25
**Amikacin**	40	1.25	2.5	2.5

## Data Availability

The original contributions presented in this study are included in the article/Supplementary Materials. Further inquiries can be directed to the corresponding author.

## Supplementary Materials

The following supporting information can be downloaded at: https://www.mdpi.com/article/10.3390/ijms262311712/s1. Reference [73] are cited in the supplementary materials.
