# Supplementary material for: 5-Substituted 4-Thiouridines, 4-Thio-2′-deoxyuridines and Their Oligoglycol Carbonate Prodrugs as Promising Antimicrobial Agents
Source: Int J Mol Sci. 2025 Dec 3;26(23):11712. doi: 10.3390/ijms262311712 (PMC12692488; doi:10.3390/ijms262311712)
Supplement: Supplementary file 1 [file ijms-26-11712-s001.zip › ijms-4010534-supplementary.pdf]

5-Substituted 4-thiouridines, 4-thio-2′-deoxyuridines and their oligoglycol carbonate prodrugs as promising antimicrobial agents

Dmitry A. Makarov[1]*, Maxim V. Jasko[1], Sergey D. Negrya[1], Inna L. Karpenko[1], Elizabeth V. Urbina[1], Vladimir O. Chekhov[1], Olga V. Efremenkova[2], Byazilya F. Vasilyeva[2], Danila V. Zimenkov[1], Anastasia I. Ushtanit[1], Sergey N. Kochetkov[1] and Liudmila A. Alexandrova[1]

[1] *Engelhardt Institute of Molecular Biology RAS, Vavilov str. 32, 119991 Moscow, Russia;*

[2] *Gause Institute of New Antibiotics, Bol'shaya Pirogovskaya str.11, Moscow, 119867, Russia*

Table of contents

NMR spectra of new compounds	Page 2
Mass spectra of new compounds	Page 18
Figure S1 - The structure of 5-dodecyloxymethyl-4-thio-dUMP - ThyX complex	Page 29
Figure S2 - Amino acid residues in contact with 5-dodecyloxymethyl-4-thio-dUMP	Page 29
Table S1 - Cytotoxicity of synthesized compounds	Page 30

NMR spectra of new compounds

5a, ^{1}H

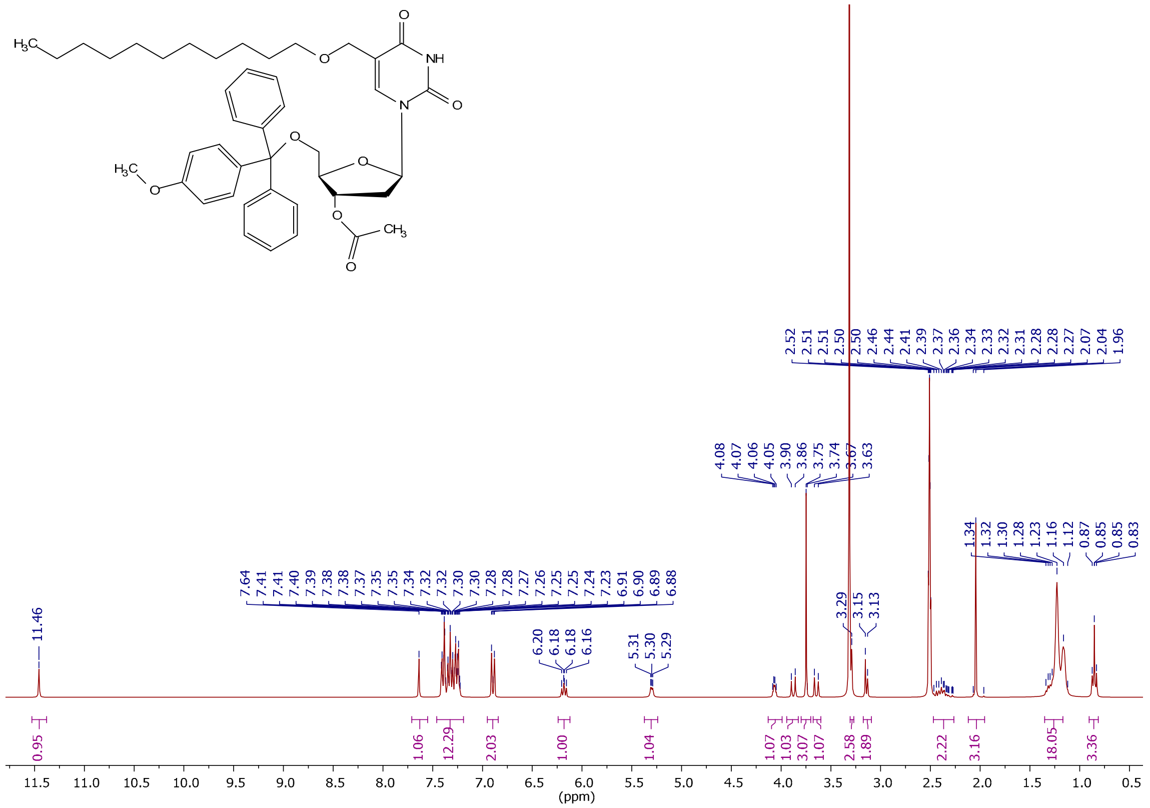

5b, ^{1}H

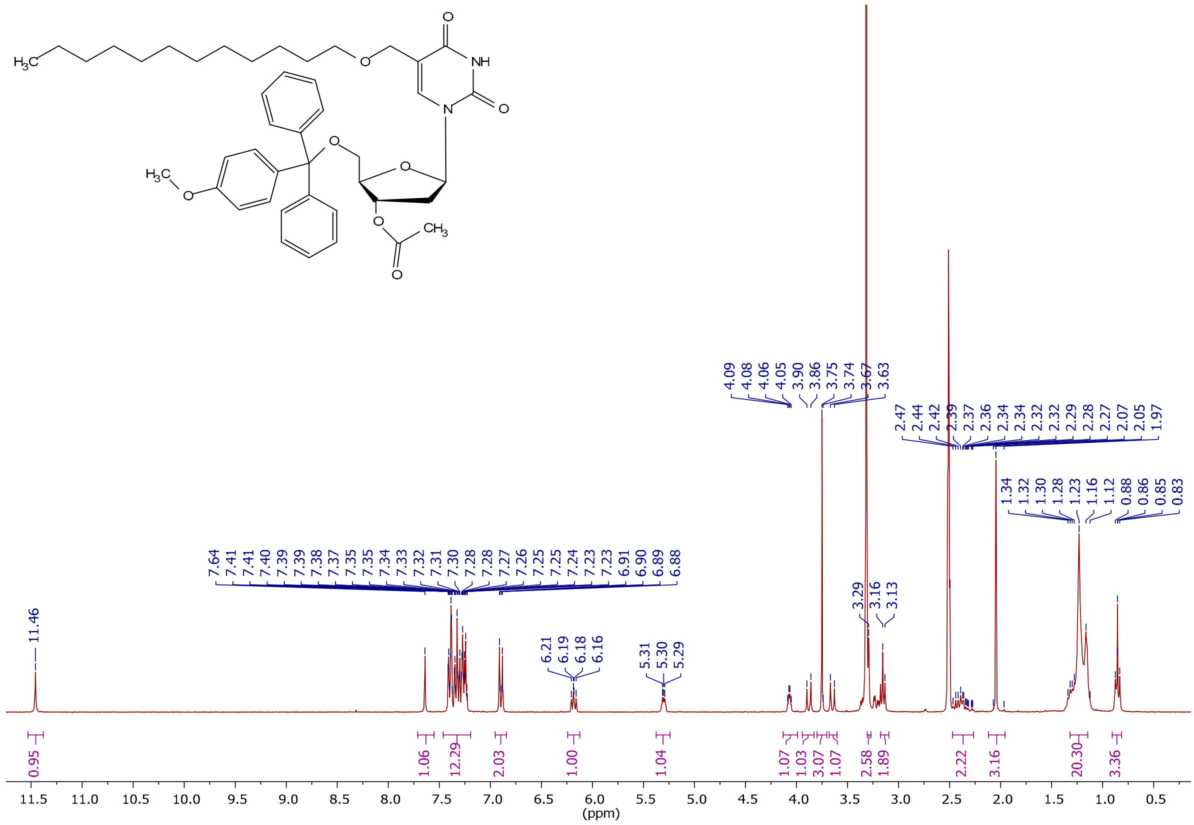

5c, ^1H

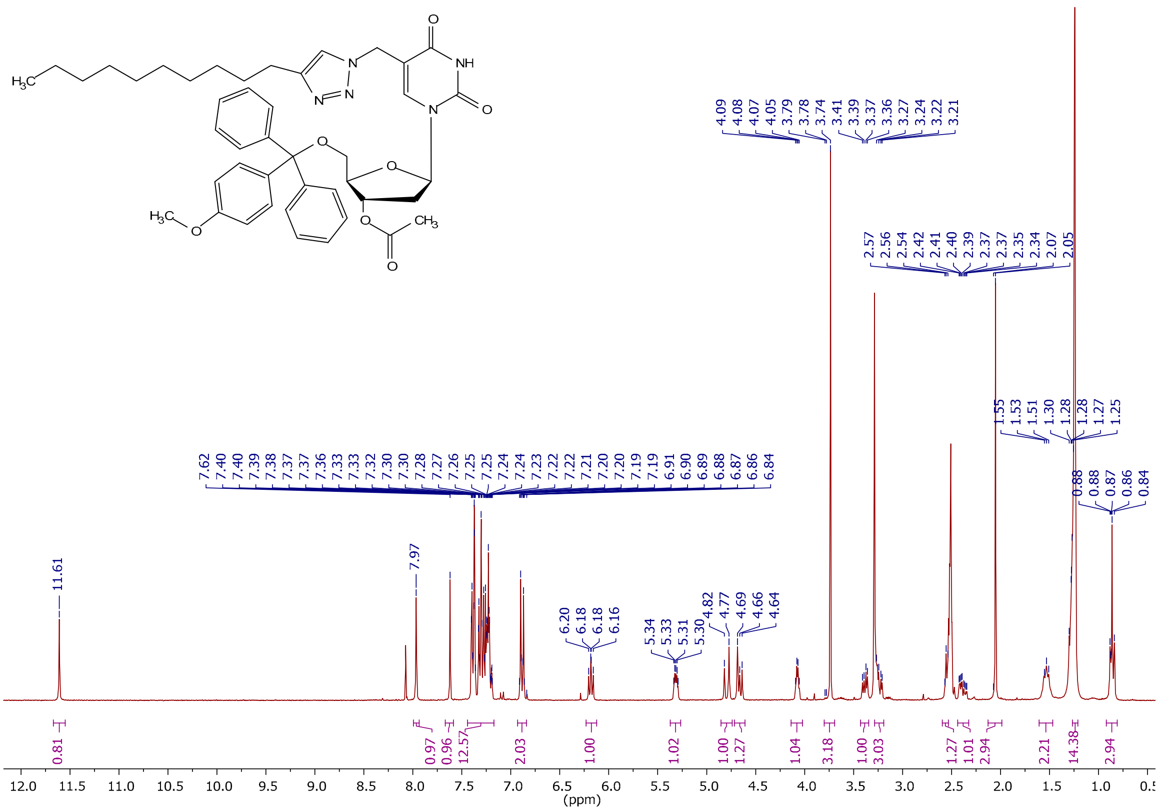

5d, ^1H

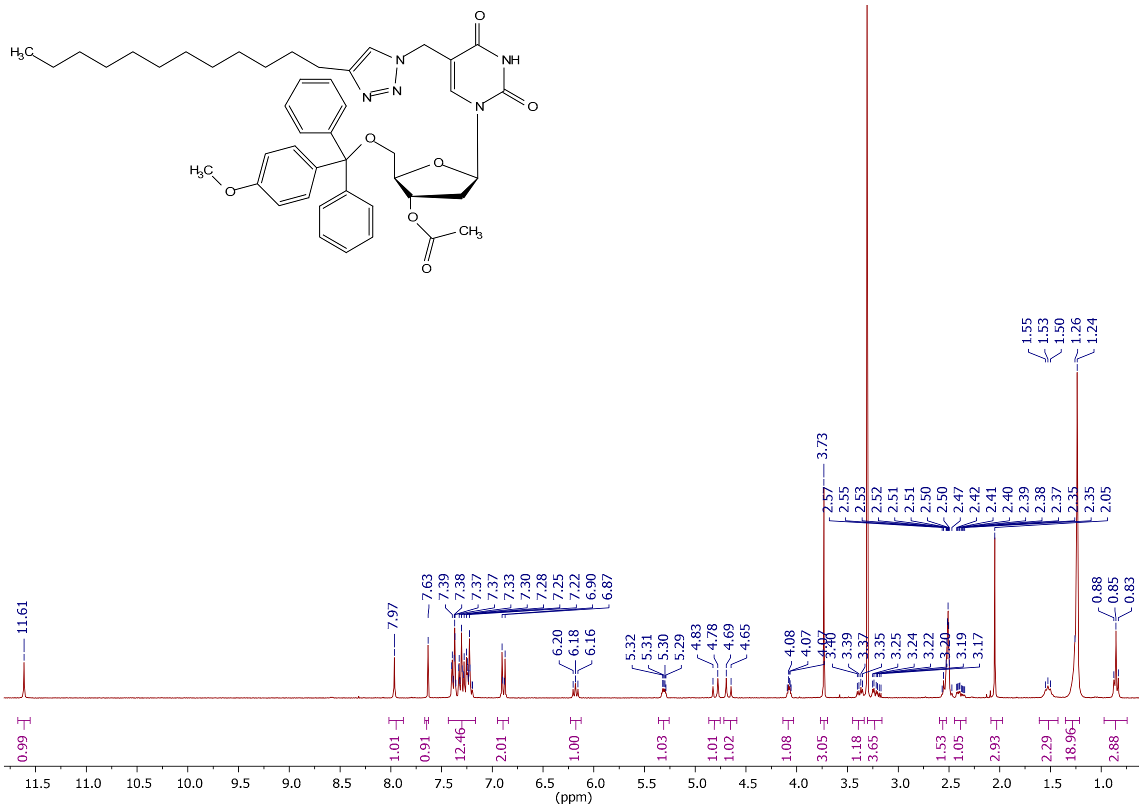

6a, ^{1}H

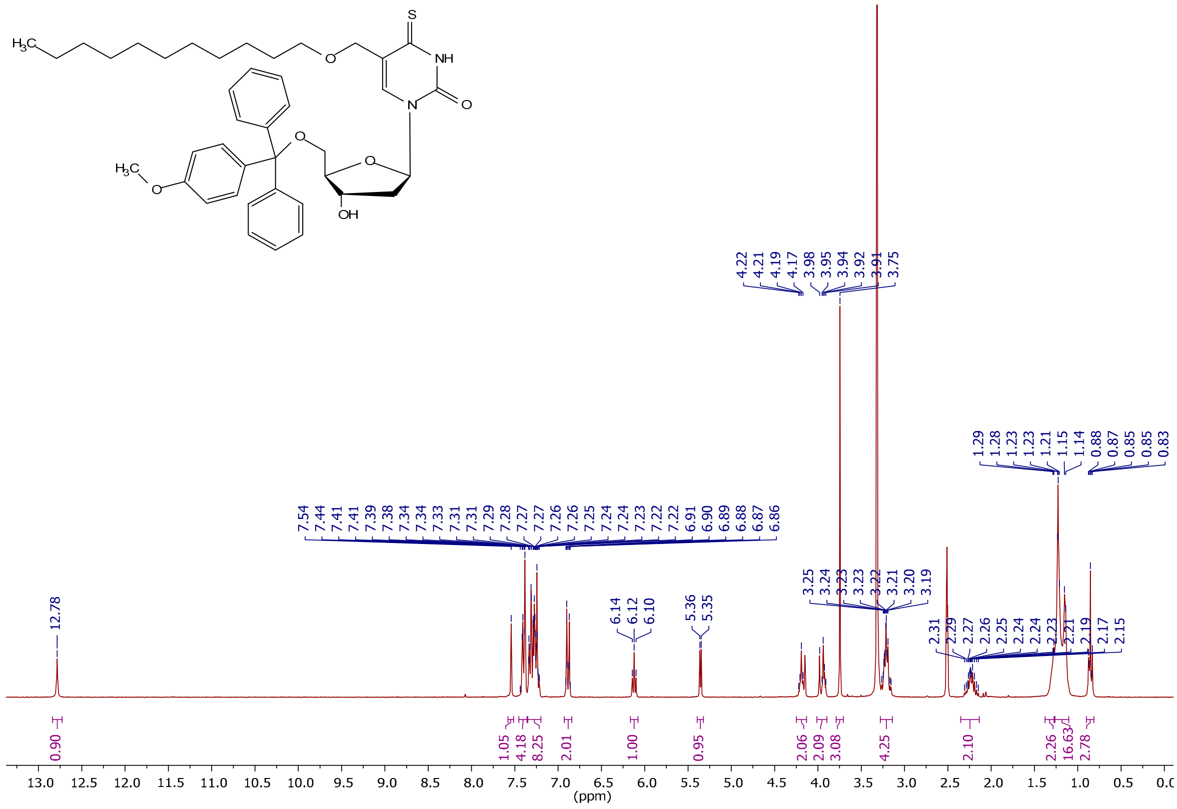

6b, ^{1}H

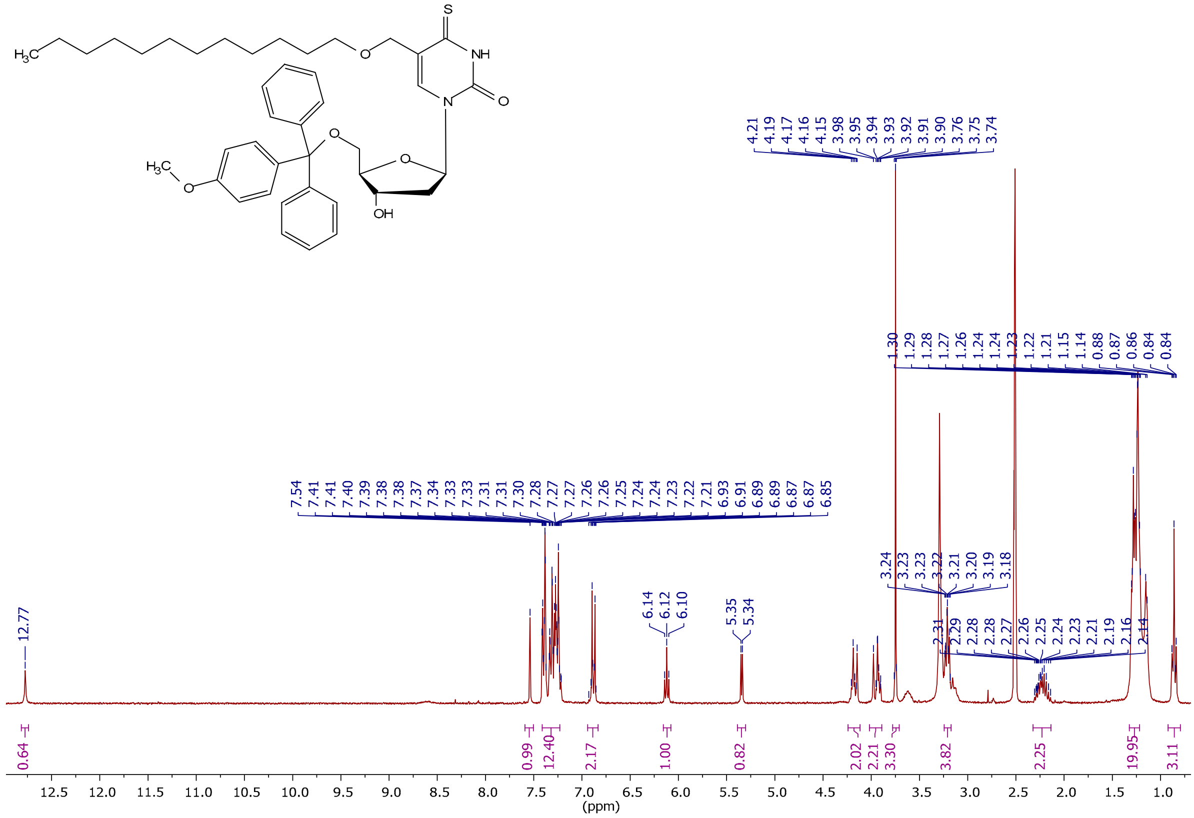

6c, ^{1}H

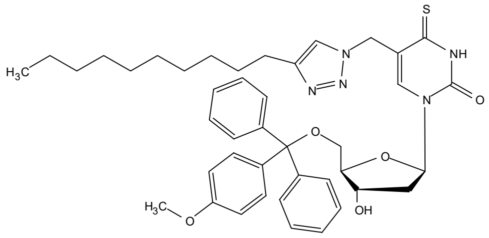

S
NH
O
N
N
N
N
O
O
OH
H3C
H3C

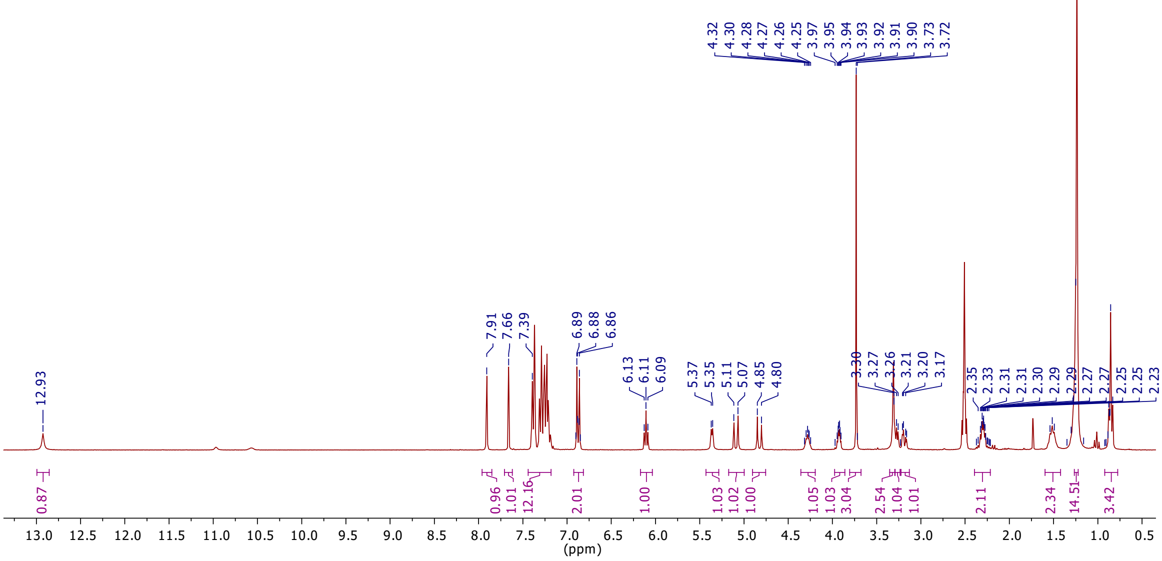

1.54 1.52 1.49 1.35 1.30 1.28 1.25 1.24 1.16 0.92 0.91 0.89 0.88 0.87 0.86 0.85 0.83
4.32 4.30 4.28 4.27 4.26 4.25 3.97 3.95 3.94 3.93 3.92 3.91 3.90 3.73 3.72
12.93
7.91 7.66 7.39
6.89 6.88 6.86
6.13 6.11 6.09
5.37 5.35 5.11 5.07 4.85 4.80
3.30 3.27 3.26 3.21 3.20 3.17
2.35 2.33 2.31 2.31 2.30 2.29 2.29 2.27 2.27 2.25 2.25 2.23
0.87 0.96 1.01 12.16 2.01 1.00 1.03 1.02 1.00 1.05 1.03 3.04 2.54 1.04 1.01 2.11 2.34 14.51 3.42
13.0 12.5 12.0 11.5 11.0 10.5 10.0 9.5 9.0 8.5 8.0 7.5 7.0 6.5 6.0 5.5 5.0 4.5 4.0 3.5 3.0 2.5 2.0 1.5 1.0 0.5
(ppm)

6d, ^{1}H

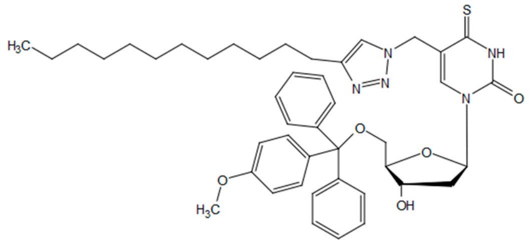

S
NH
O
N
N
N
N
O
O
OH
H3C
H3C

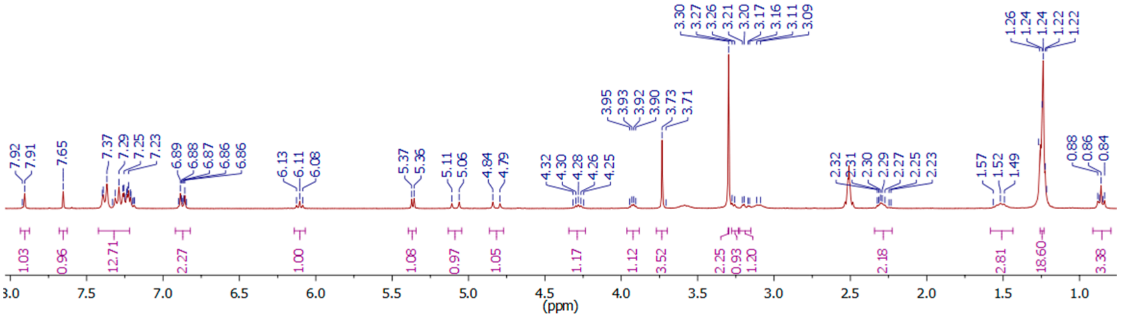

3.30 3.27 3.26 3.21 3.20 3.17 3.16 3.11 3.09
1.26 1.24 1.24 1.22 1.22
3.95 3.93 3.92 3.90 3.73 3.71
7.92 7.91 7.65 7.37 7.29 7.25 7.23 6.89 6.88 6.87 6.86 6.86
6.13 6.11 6.08 5.37 5.36 5.11 5.06 4.84 4.79
4.32 4.30 4.28 4.26 4.25
2.32 2.31 2.30 2.29 2.27 2.25 2.23
1.57 1.52 1.49
0.88 0.86 0.84
1.03 0.96 12.71 2.27 1.00 1.08 0.97 1.05 1.17 1.12 3.52 2.25 0.93 1.20 2.18 2.81 18.60 3.38
8.0 7.5 7.0 6.5 6.0 5.5 5.0 4.5 4.0 3.5 3.0 2.5 2.0 1.5 1.0
(ppm)

3a, ^1H

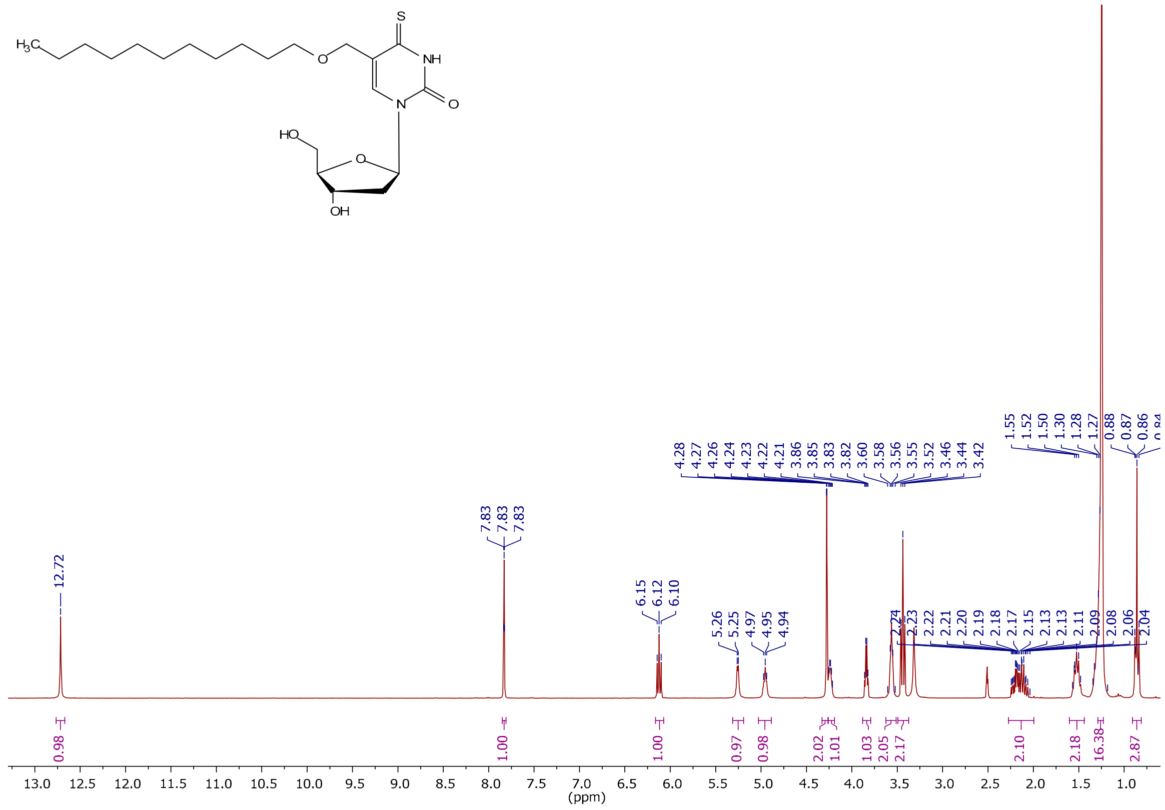

12.72
7.83
7.83
7.83
6.15
6.12
6.10
5.26
5.25
4.97
4.95
4.94
4.28
4.27
4.26
4.24
4.23
4.22
4.21
3.86
3.85
3.83
3.82
3.60
3.58
3.56
3.55
3.52
3.46
3.44
3.42
2.24
2.23
2.22
2.21
2.20
2.19
2.18
2.17
2.15
2.13
2.13
2.11
2.09
2.08
2.06
2.04
1.55
1.52
1.50
1.30
1.28
1.27
0.88
0.87
0.86
0.98
1.00
1.00
0.97
0.98
2.02
1.01
1.03
2.05
2.17
2.10
2.18
16.38
2.87
13.0 12.5 12.0 11.5 11.0 10.5 10.0 9.5 9.0 8.5 8.0 7.5 7.0 6.5 6.0 5.5 5.0 4.5 4.0 3.5 3.0 2.5 2.0 1.5 1.0
(ppm)

3a, ^{13}C

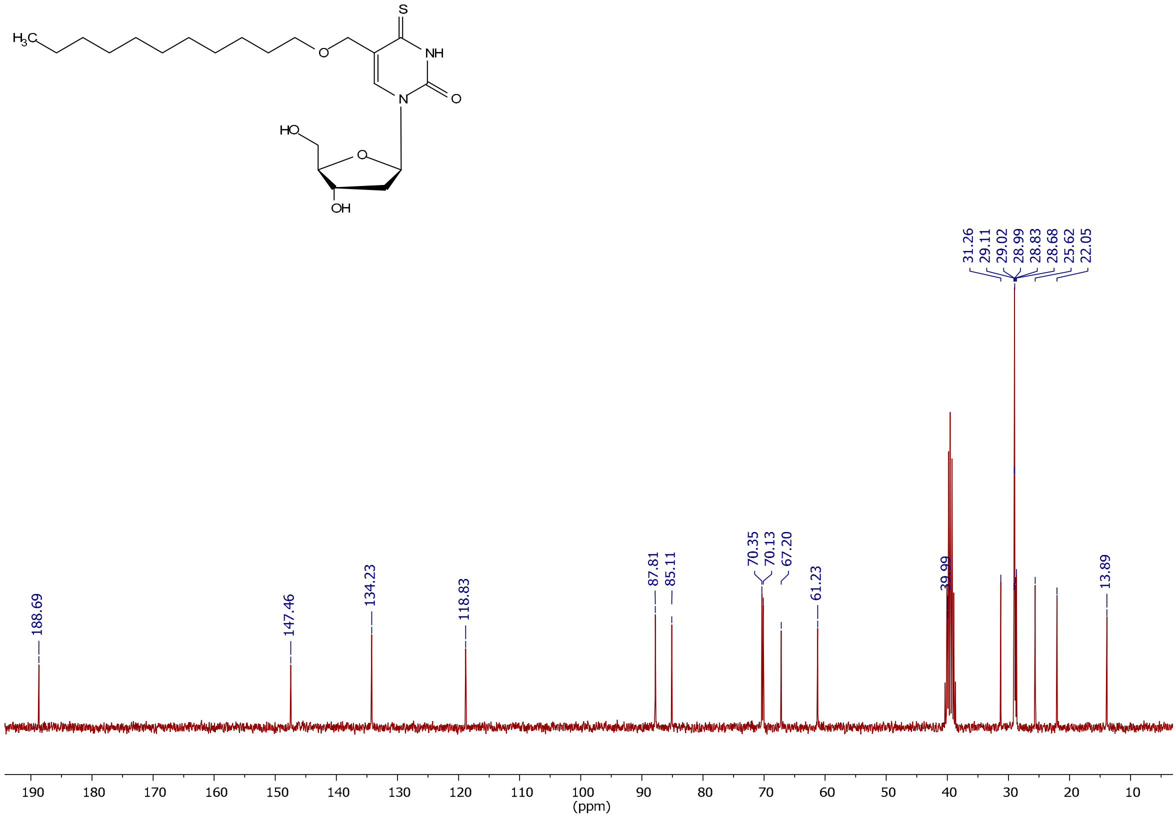

188.69
147.46
134.23
118.83
87.81
85.11
70.35
70.13
67.20
61.23
39.99
31.26
29.11
29.02
28.99
28.83
28.68
25.62
22.05
13.89
190 180 170 160 150 140 130 120 110 100 90 80 70 60 50 40 30 20 10
(ppm)

3b, ^{1}H

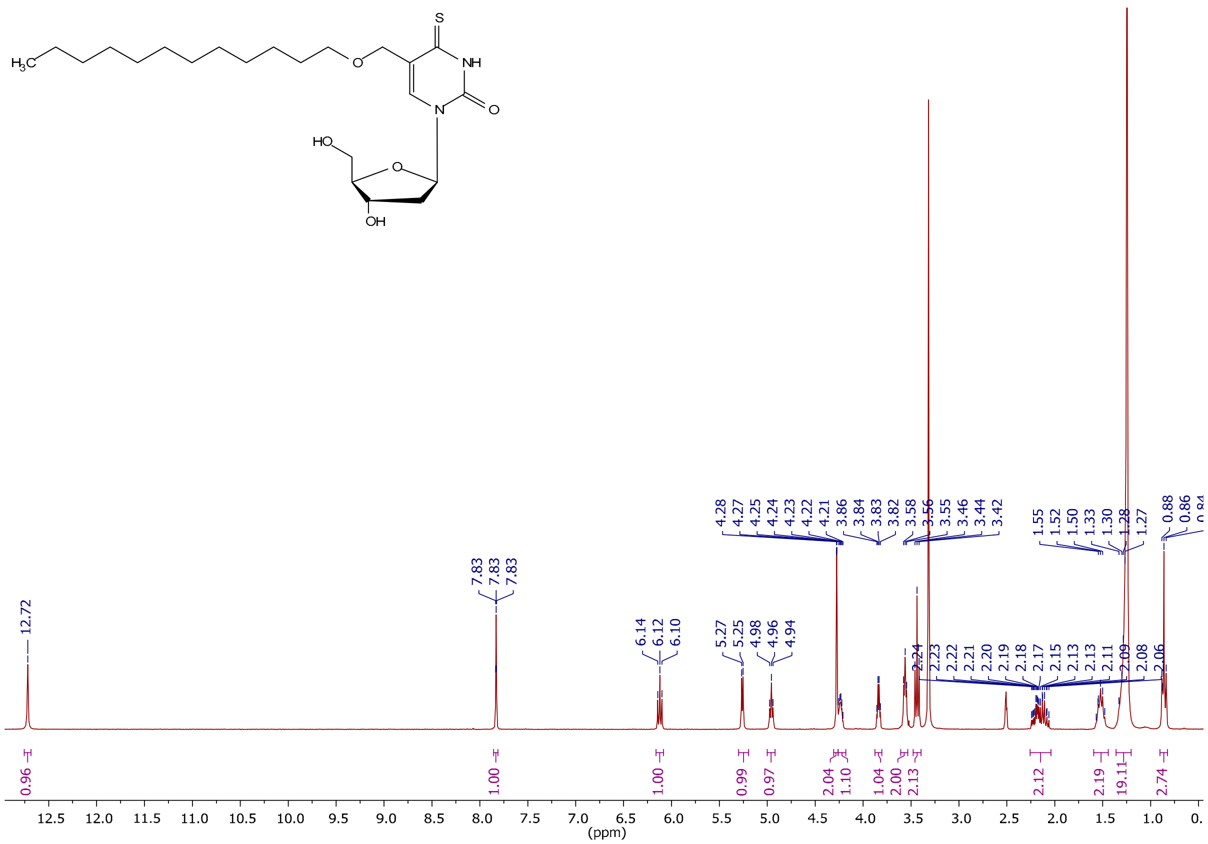

S
NH
O
N
H_3C
O
HO
O
OH
12.72
7.83
7.83
7.83
6.14
6.12
6.10
5.27
5.25
4.98
4.96
4.94
4.28
4.27
4.25
4.24
4.23
4.22
4.21
3.86
3.84
3.83
3.82
3.58
3.56
3.55
3.46
3.44
3.42
2.24
2.23
2.22
2.21
2.20
2.19
2.18
2.17
2.15
2.13
2.13
2.11
2.09
2.08
2.06
1.55
1.52
1.50
1.33
1.30
1.28
1.27
0.88
0.86
0.96
1.00
1.00
0.99
0.97
2.04
1.10
1.04
2.00
2.13
2.12
2.19
19.11
2.74
12.5 12.0 11.5 11.0 10.5 10.0 9.5 9.0 8.5 8.0 7.5 7.0 6.5 6.0 5.5 5.0 4.5 4.0 3.5 3.0 2.5 2.0 1.5 1.0
(ppm)

3b, ^{13}C

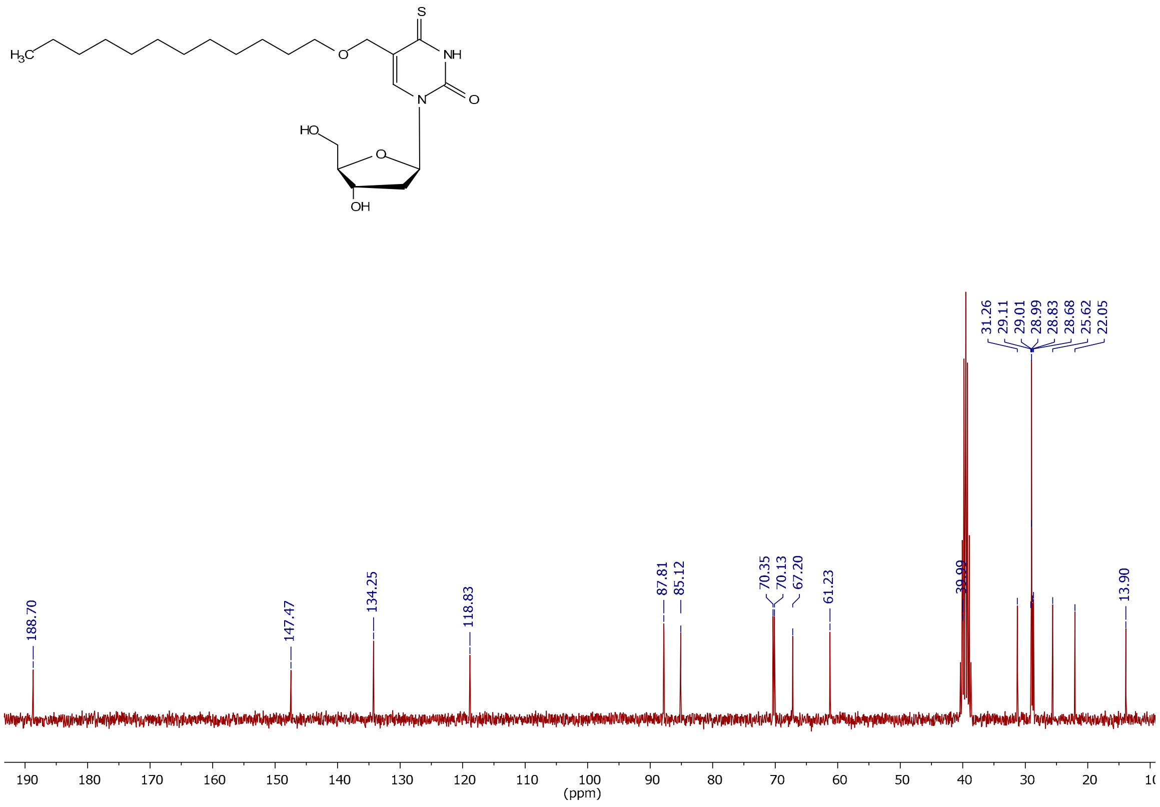

S
NH
O
N
H_3C
O
HO
O
OH
188.70
147.47
134.25
118.83
87.81
85.12
70.35
70.13
67.20
61.23
39.99
31.26
29.11
29.01
28.99
28.83
28.68
25.62
22.05
13.90
190 180 170 160 150 140 130 120 110 100 90 80 70 60 50 40 30 20
(ppm)

3c, ^{1}H

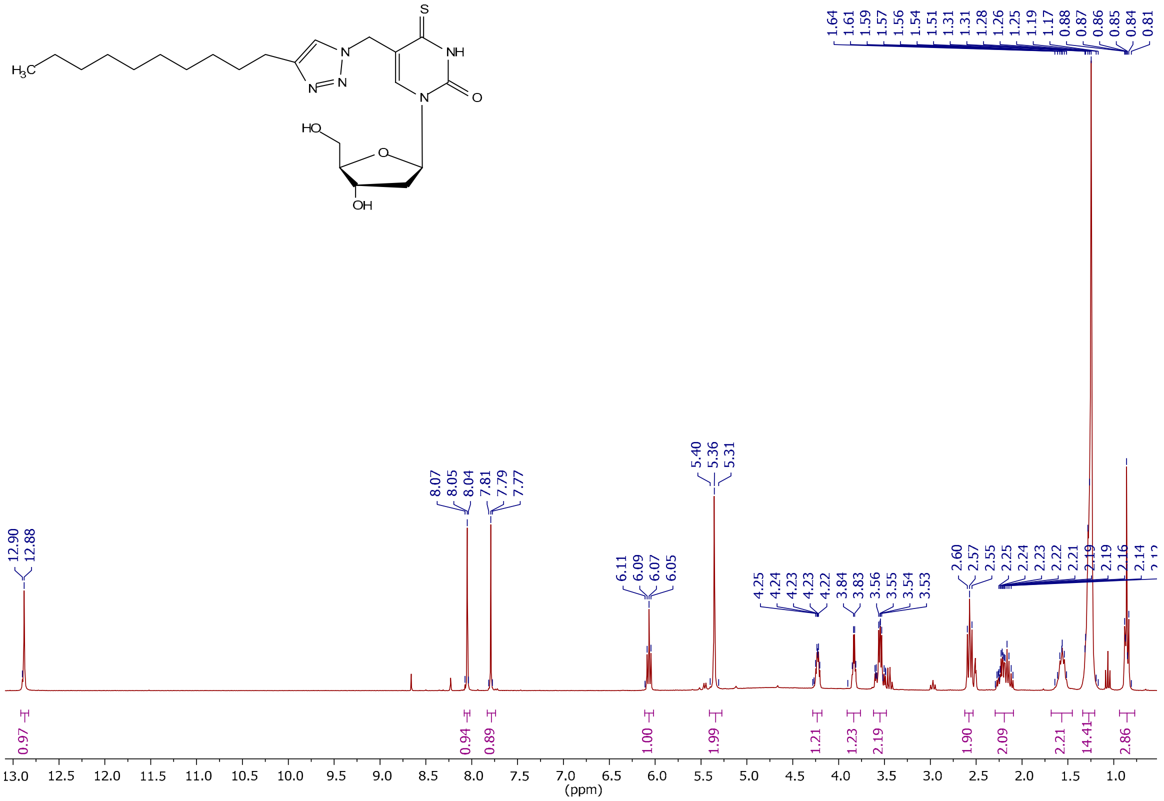

H3C
S
NH
N
O
HO
OH
1.64 1.61 1.59 1.57 1.56 1.54 1.51 1.31 1.31 1.28 1.26 1.25 1.19 1.17 0.88 0.87 0.86 0.85 0.84 0.81
12.90 12.88
8.07 8.05 8.04 7.81 7.79 7.77
6.11 6.09 6.07 6.05
5.40 5.36 5.31
4.25 4.24 4.23 4.23 4.22 3.84 3.83 3.56 3.55 3.54 3.53
2.60 2.57 2.55 2.25 2.24 2.23 2.22 2.21 2.19 2.19 2.16 2.14
0.97 0.94 0.89 1.00 1.99 1.21 1.23 2.19 1.90 2.09 2.21 14.41 2.86
13.0 12.5 12.0 11.5 11.0 10.5 10.0 9.5 9.0 8.5 8.0 7.5 7.0 6.5 6.0 5.5 5.0 4.5 4.0 3.5 3.0 2.5 2.0 1.5 1.0
(ppm)

3c, ^{13}C

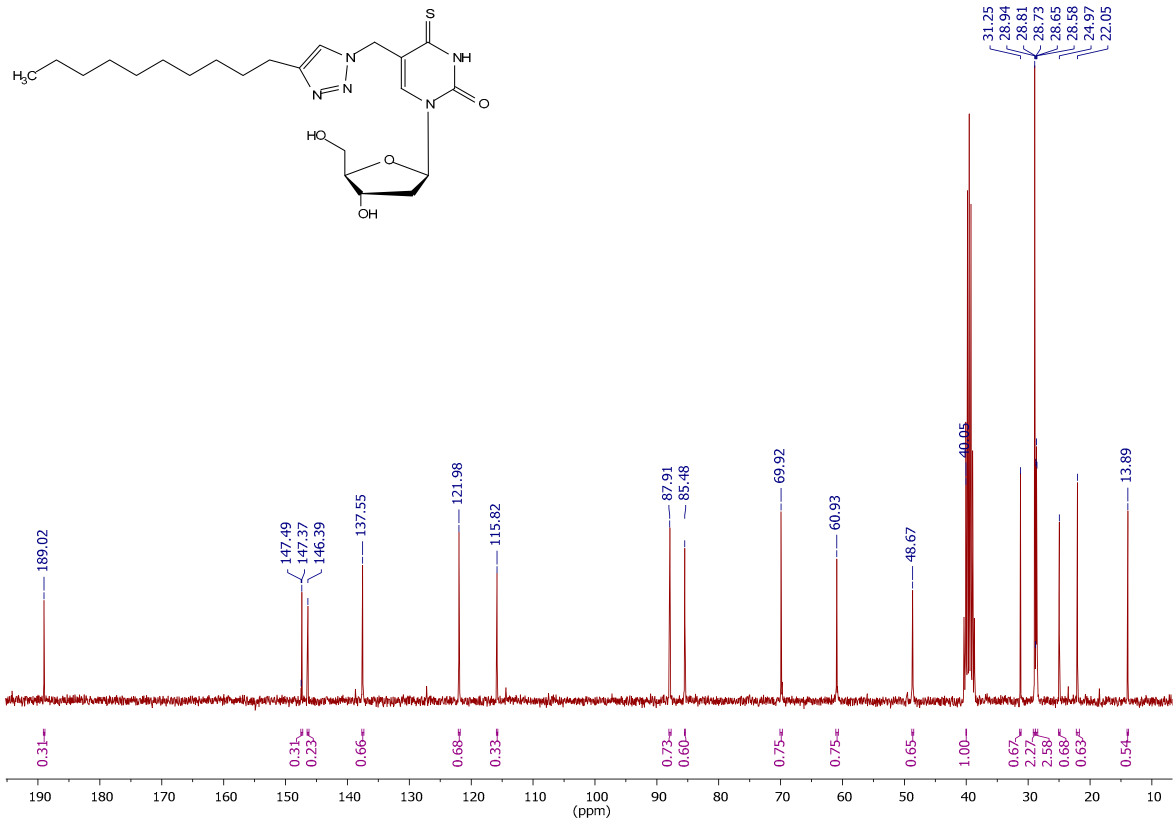

H3C
S
NH
N
O
HO
OH
31.25 28.94 28.81 28.73 28.65 28.58 24.97 22.05
189.02
147.49 147.37 146.39
137.55
121.98
115.82
87.91 85.48
69.92
60.93
48.67
40.05
13.89
0.31 0.31 0.23 0.66 0.68 0.33 0.73 0.60 0.75 0.75 0.65 1.00 0.67 2.27 2.58 0.68 0.63 0.54
190 180 170 160 150 140 130 120 110 100 90 80 70 60 50 40 30 20 10
(ppm)

3d, ^{1}H

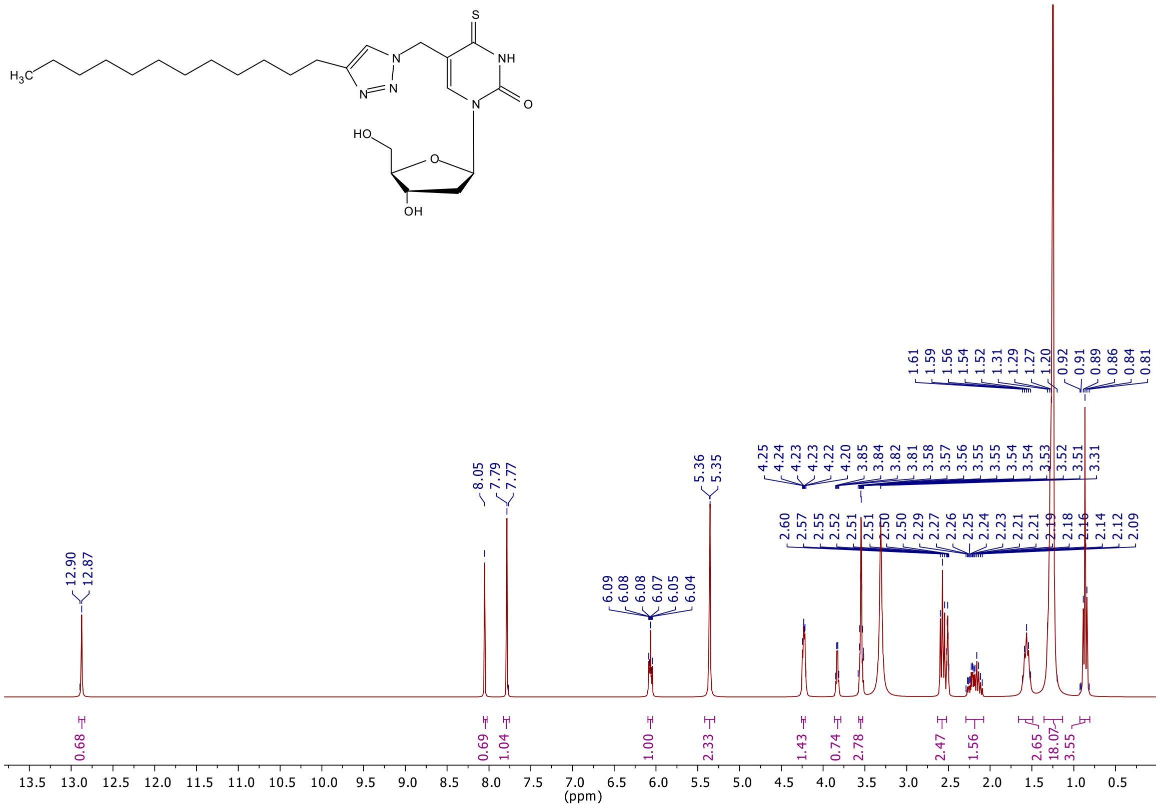

3d, ^{13}C

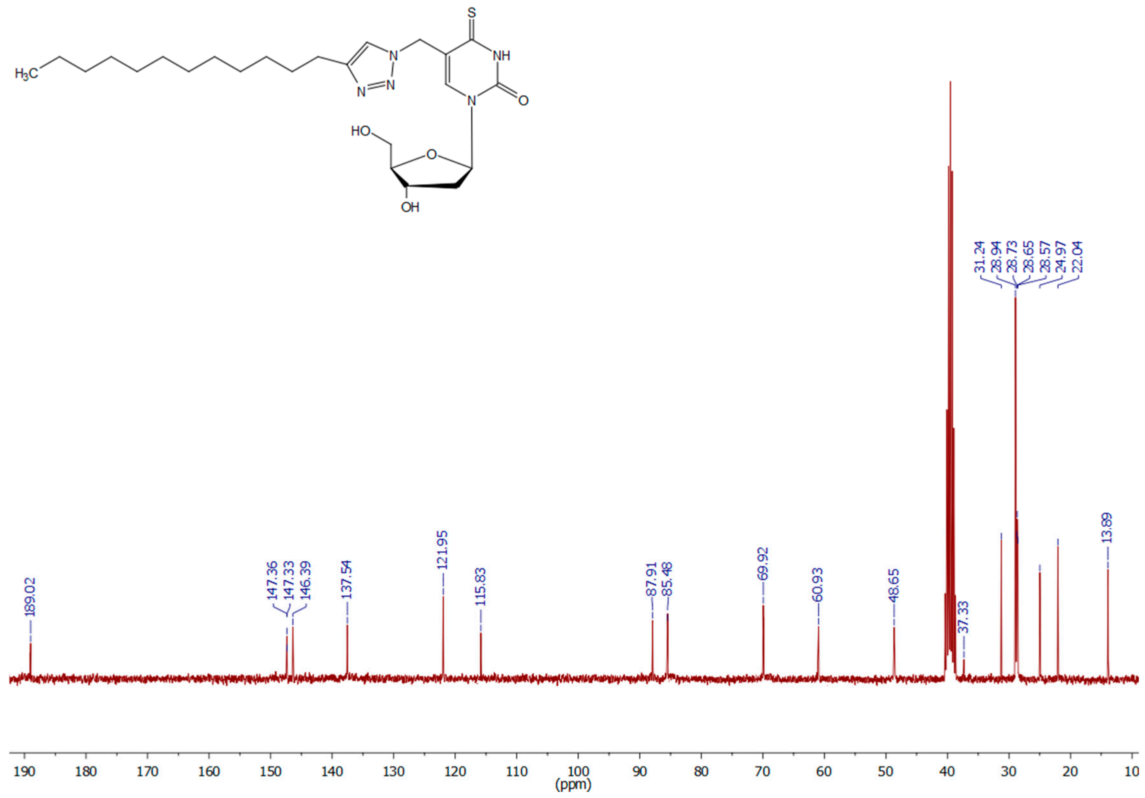

4a, ^{1}H

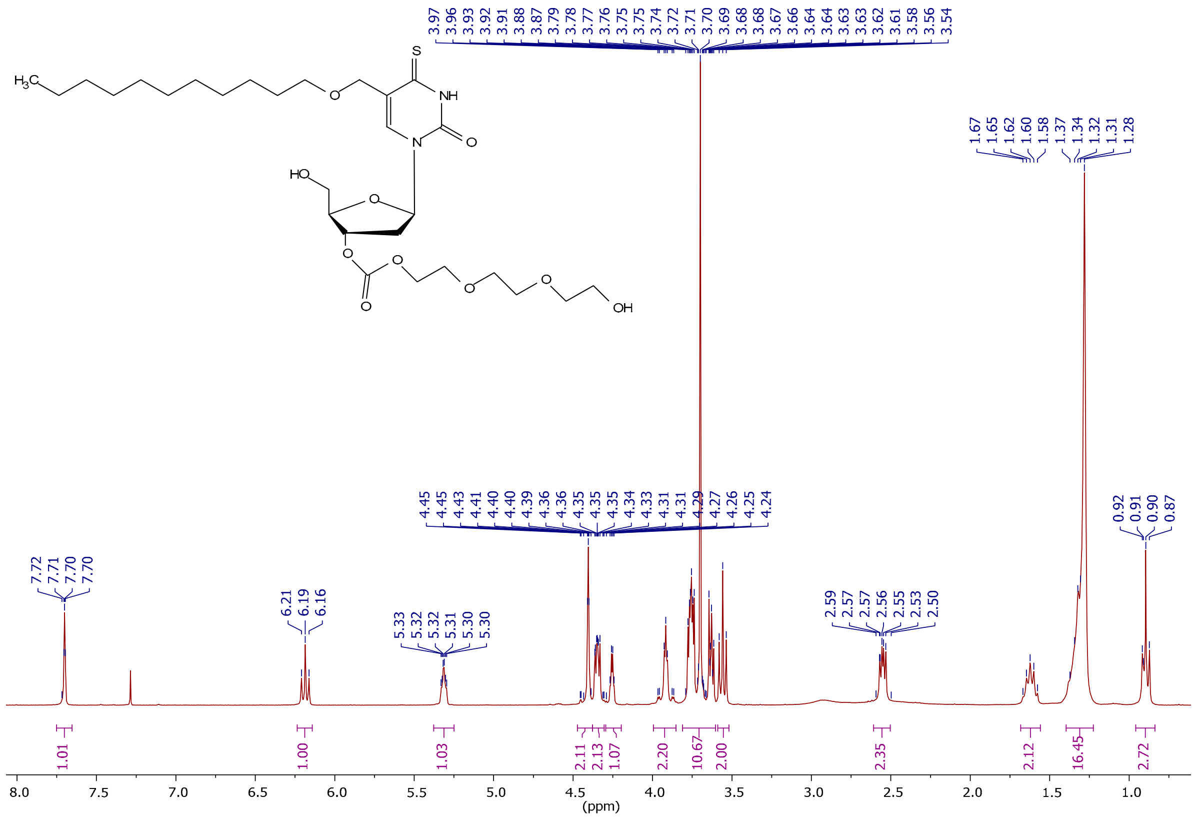

4a, ^{13}C

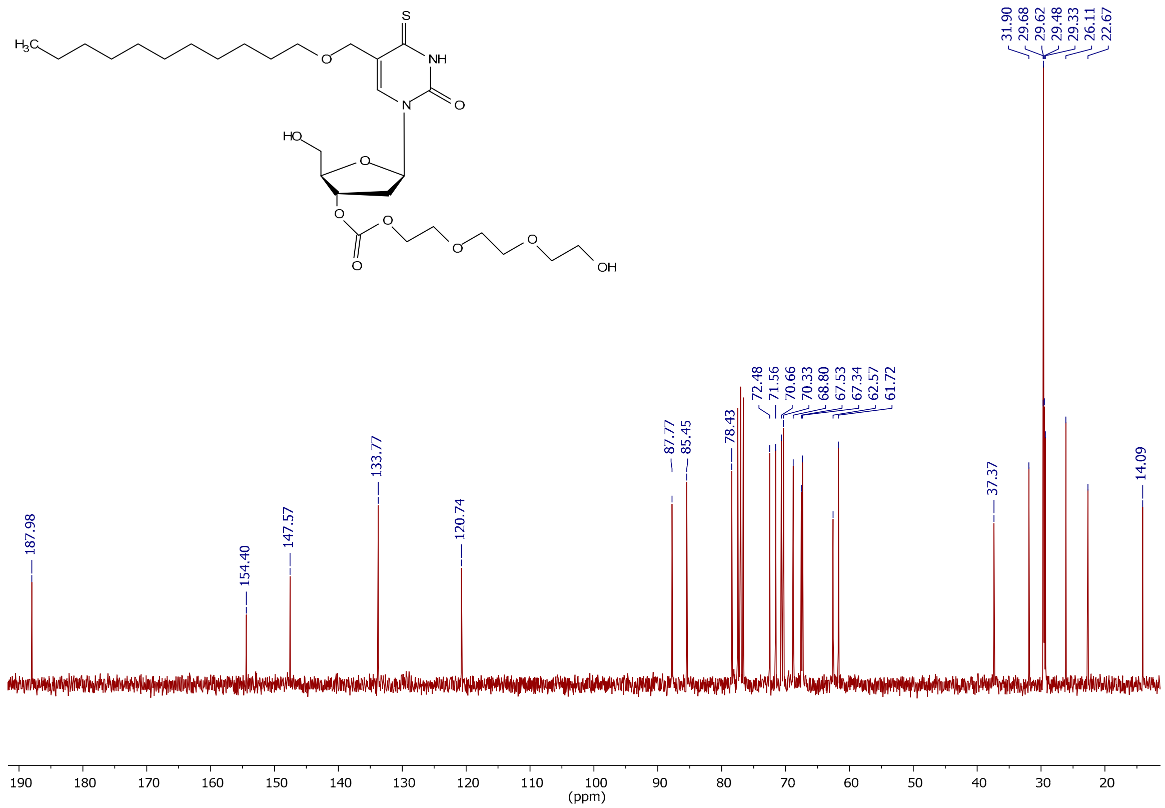

4b, ^{1}H

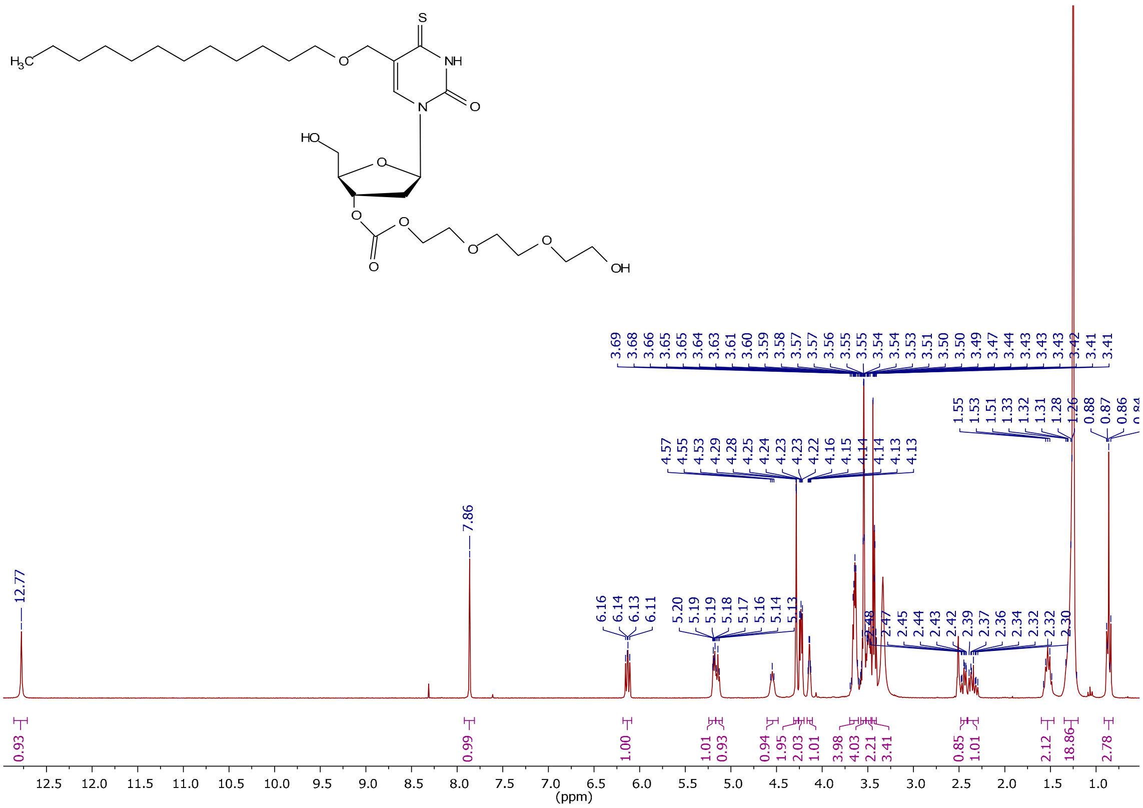
S
NH
O
N
H_3C
O
HO
O
O
O
O
O
O
OH
12.77
7.86
6.16 6.14 6.13 6.11
5.20 5.19 5.19 5.18 5.17 5.16 5.14 5.13
4.57 4.55 4.53 4.29 4.28 4.25 4.24 4.23 4.23 4.22 4.16 4.15 4.14 4.14 4.13 4.13
3.69 3.68 3.66 3.65 3.65 3.64 3.63 3.61 3.60 3.59 3.58 3.57 3.57 3.56 3.55 3.55 3.54 3.54 3.53 3.51 3.50 3.50 3.49 3.47 3.44 3.43 3.43 3.43 3.42 3.41 3.41
2.48 2.47 2.45 2.44 2.43 2.42 2.39 2.37 2.36 2.34 2.32 2.32 2.30
1.55 1.53 1.51 1.33 1.32 1.31 1.28 1.26
0.88 0.87 0.86
0.93
0.99
1.00
1.01 0.93
0.94 1.95 2.03 1.01
3.98 4.03 2.21 3.41
0.85 1.01
2.12 18.86 2.78
12.5 12.0 11.5 11.0 10.5 10.0 9.5 9.0 8.5 8.0 7.5 7.0 6.5 6.0 5.5 5.0 4.5 4.0 3.5 3.0 2.5 2.0 1.5 1.0
(ppm)

4b, ^{13}C

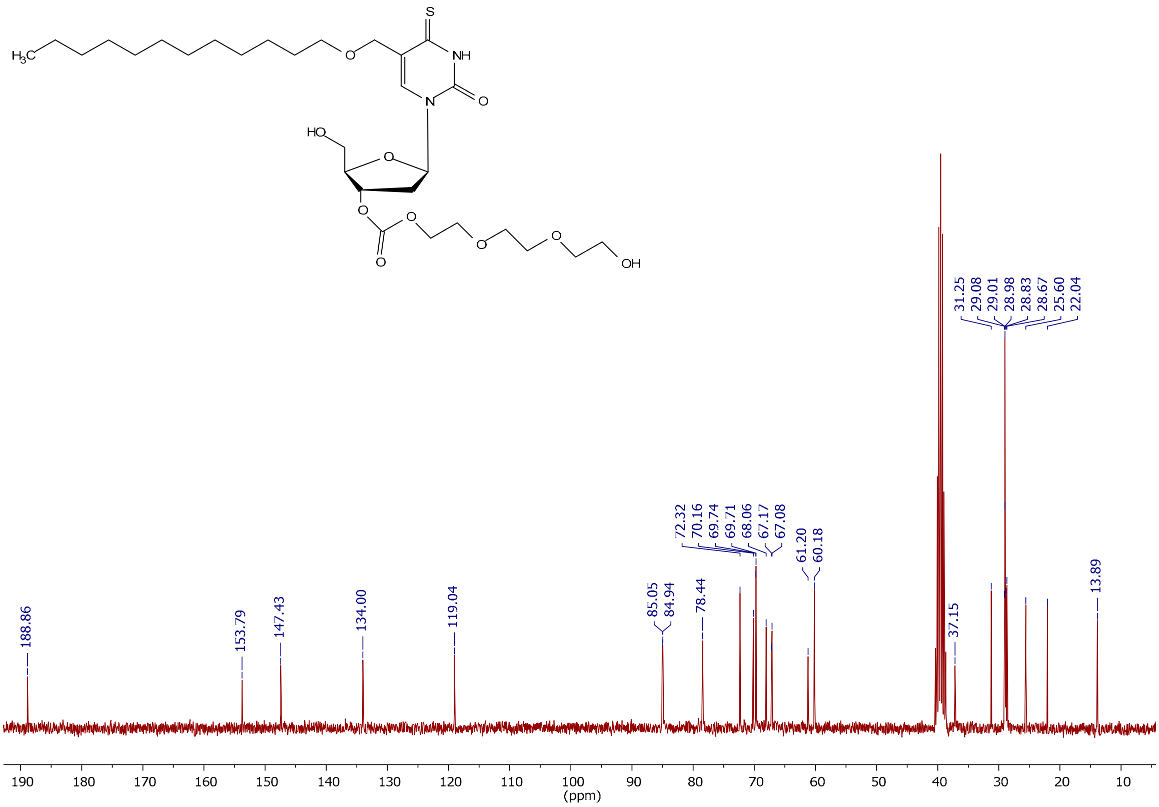
S
NH
O
N
H_3C
O
HO
O
O
O
O
O
O
OH
188.86
153.79
147.43
134.00
119.04
85.05 84.94
78.44
72.32 70.16 69.74 69.71 68.06 67.17 67.08
61.20 60.18
37.15
31.25 29.08 29.01 28.98 28.83 28.67 25.60 22.04
13.89
190 180 170 160 150 140 130 120 110 100 90 80 70 60 50 40 30 20 10
(ppm)

4c, ¹H

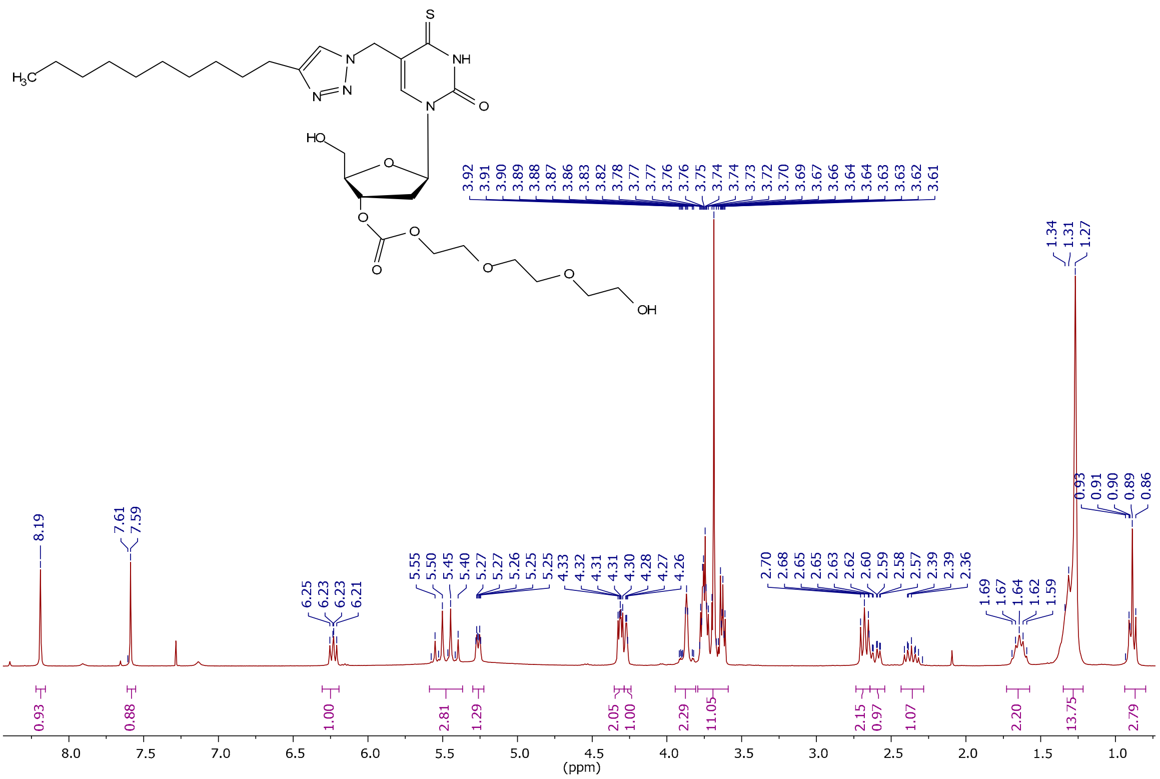
H3C
S
NH
N
N
N
N
O
HO
O
O
O
O
O
O
OH
3.92 3.91 3.90 3.89 3.88 3.87 3.86 3.83 3.82 3.78 3.77 3.77 3.76 3.75 3.74 3.74 3.73 3.72 3.70 3.69 3.67 3.66 3.64 3.64 3.63 3.63 3.62 3.61
1.34 1.31 1.27
0.93 0.91 0.90 0.89 0.86
8.19
7.61 7.59
6.25 6.23 6.23 6.21
5.55 5.50 5.45 5.40 5.27 5.27 5.26 5.25 5.25
4.33 4.32 4.31 4.31 4.30 4.28 4.27 4.26
2.70 2.68 2.65 2.65 2.63 2.62 2.60 2.59 2.58 2.57 2.39 2.39 2.36
1.69 1.67 1.64 1.62 1.59
0.93 0.88 1.00 2.81 1.29 2.05 1.00 2.29 11.05 2.15 0.97 1.07 2.20 13.75 2.79
8.0 7.5 7.0 6.5 6.0 5.5 5.0 4.5 4.0 3.5 3.0 2.5 2.0 1.5 1.0
(ppm)

4c, ¹³C

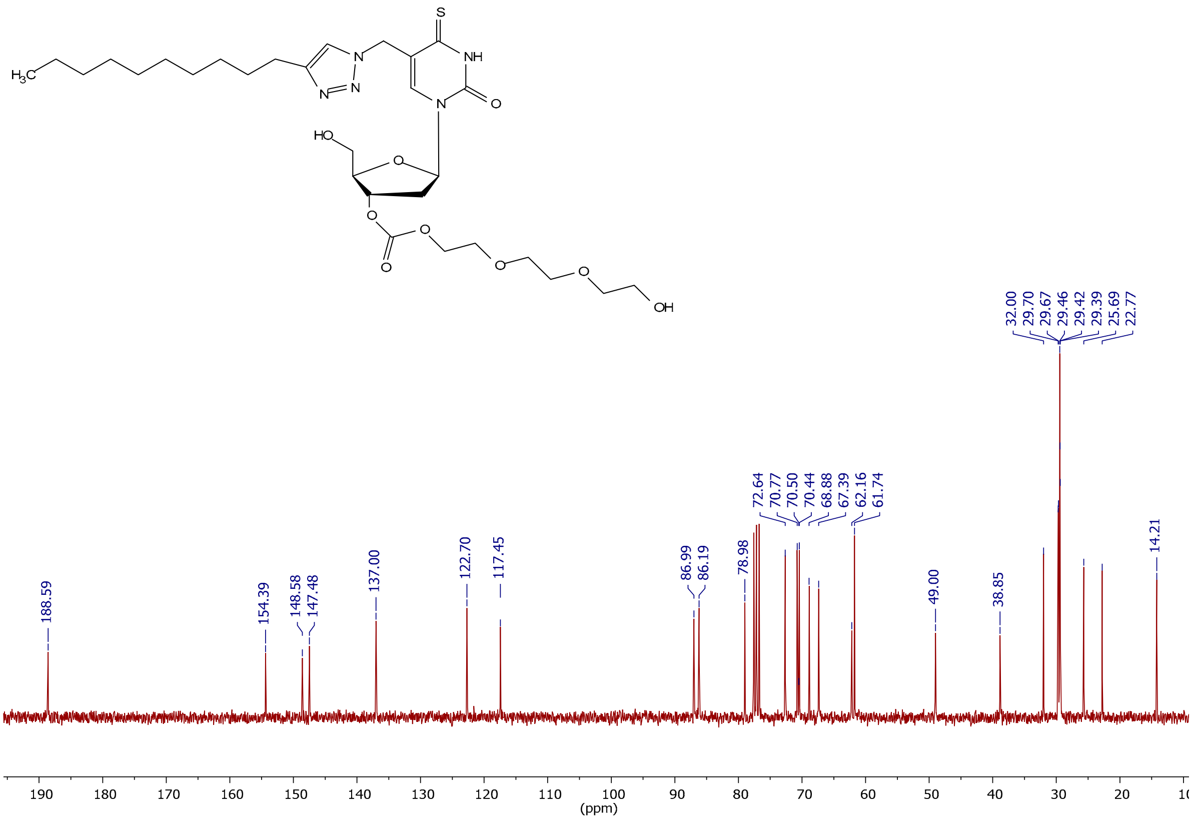
H3C
S
NH
N
N
N
N
O
HO
O
O
O
O
O
O
OH
32.00 29.70 29.67 29.46 29.42 29.39 25.69 22.77
72.64 70.77 70.50 70.44 68.88 67.39 62.16 61.74
188.59
154.39
148.58 147.48
137.00
122.70
117.45
86.99 86.19
78.98
49.00
38.85
14.21
190 180 170 160 150 140 130 120 110 100 90 80 70 60 50 40 30 20 10
(ppm)

4d, ^{1}H

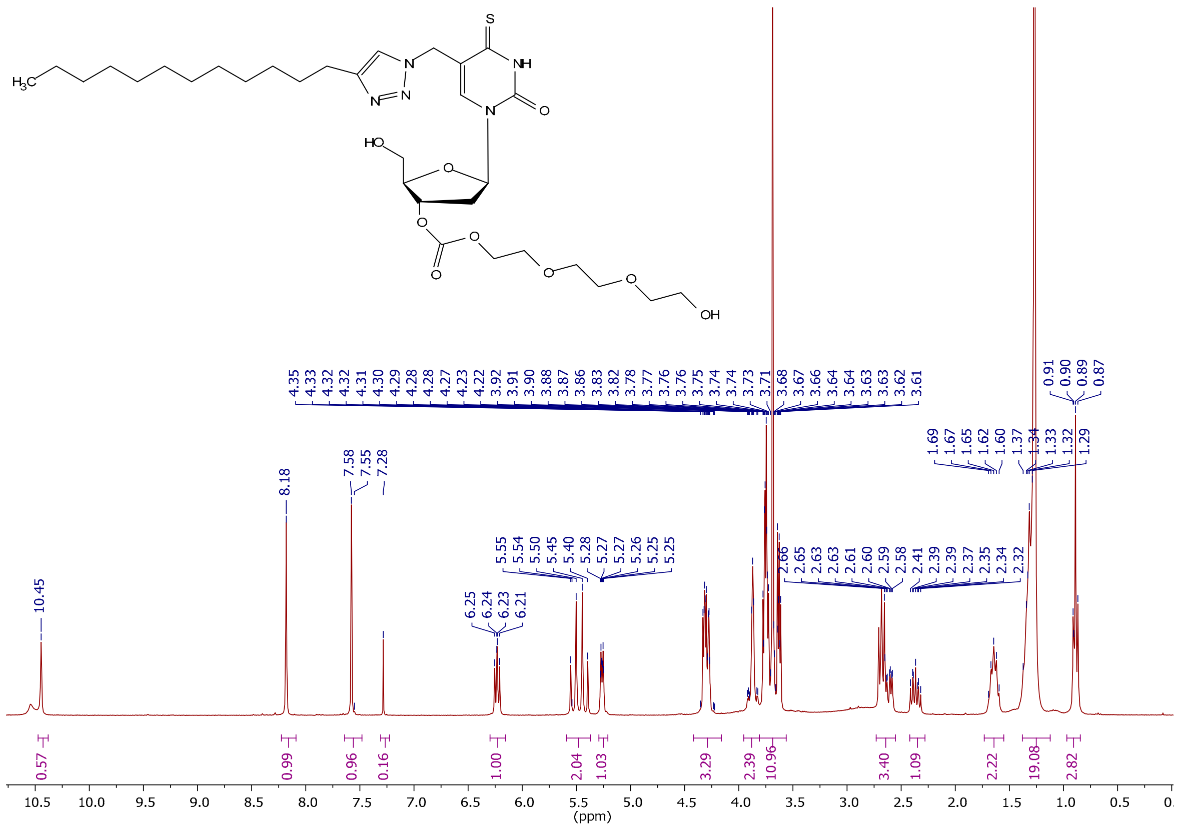

4d, ^{13}C

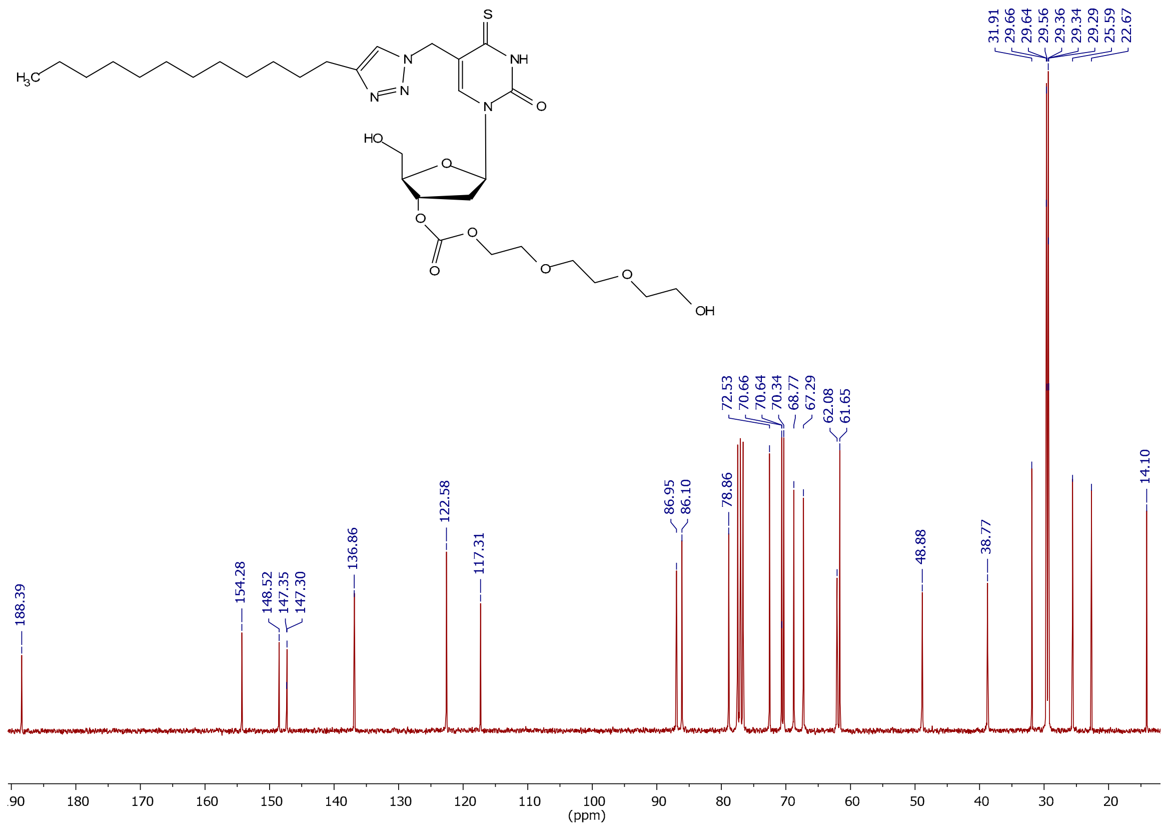

3e, ^{1}H

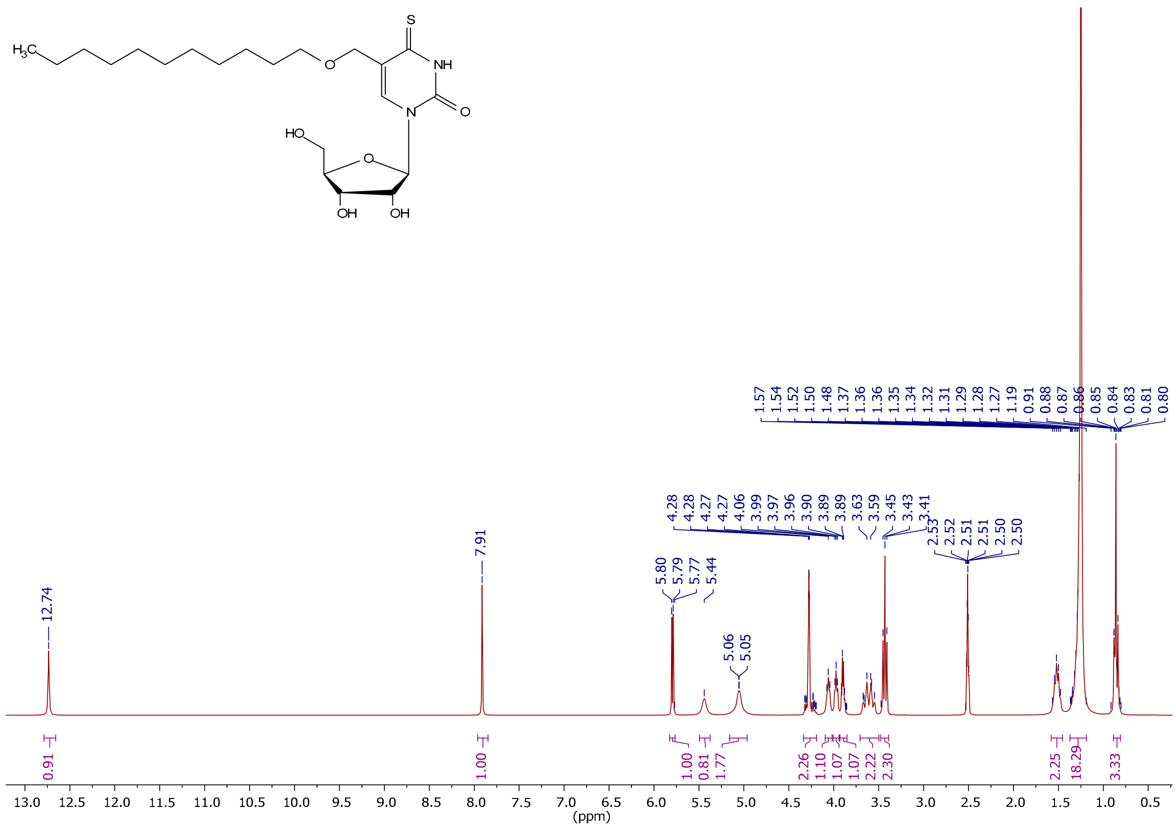

3e, ^{13}C

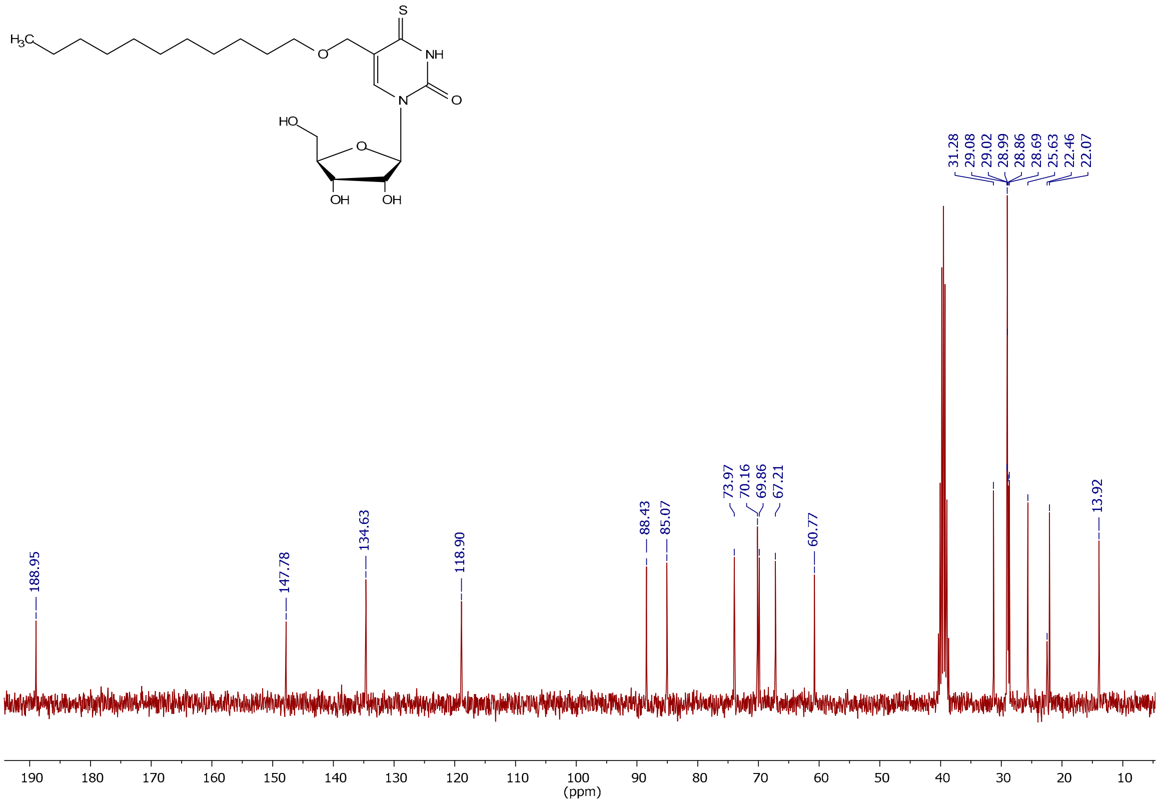

3f, ^1H

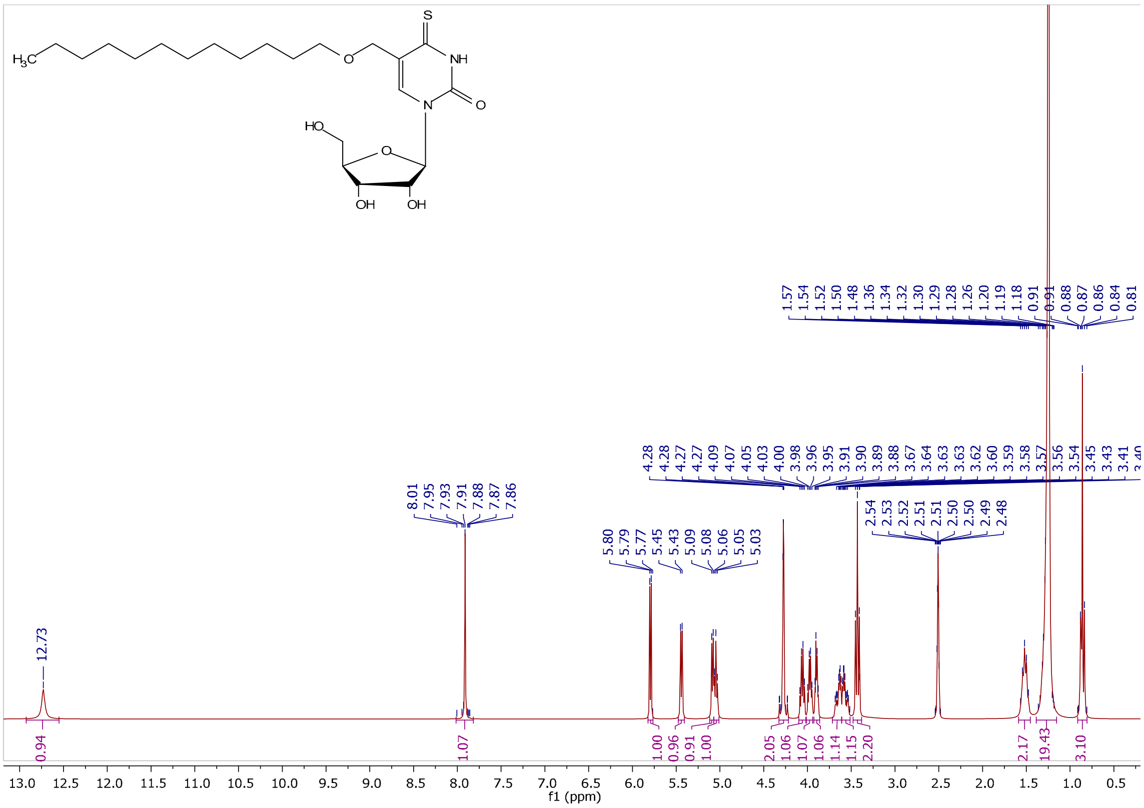

3f, ^{13}C

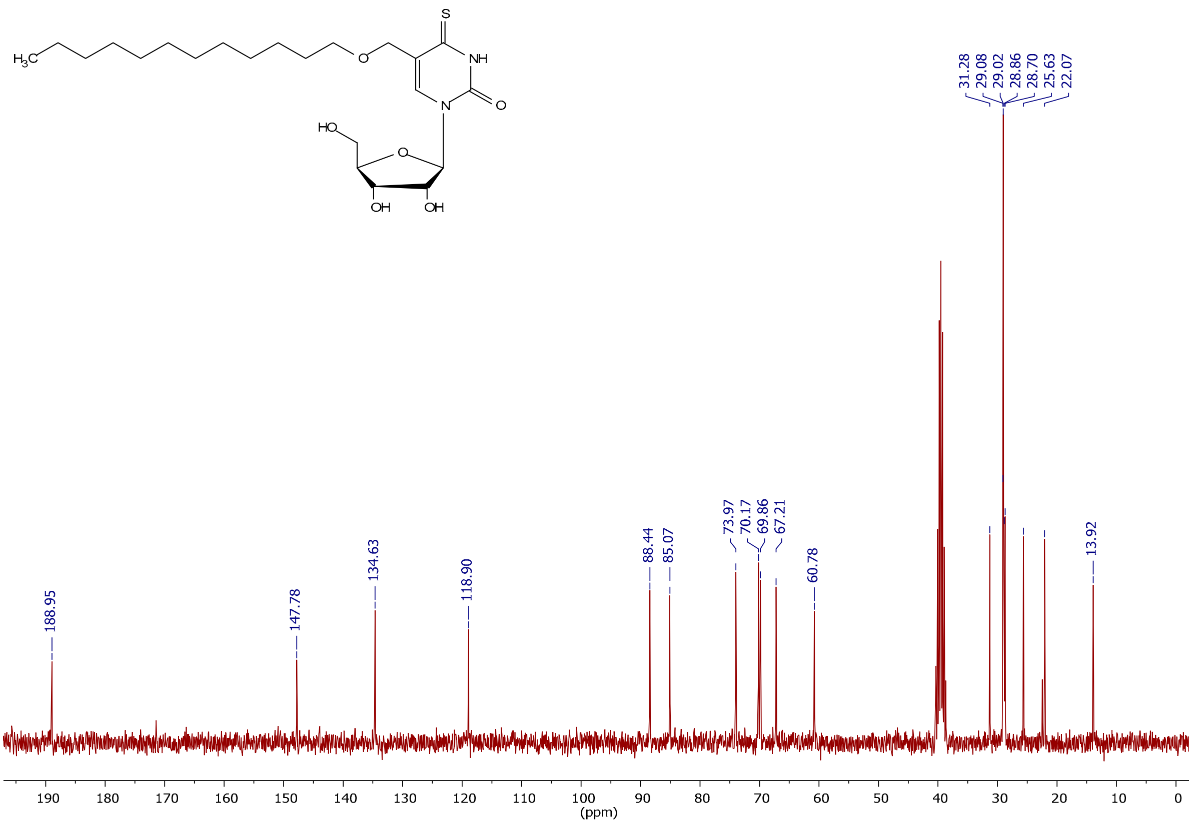

3g, ^{1}H

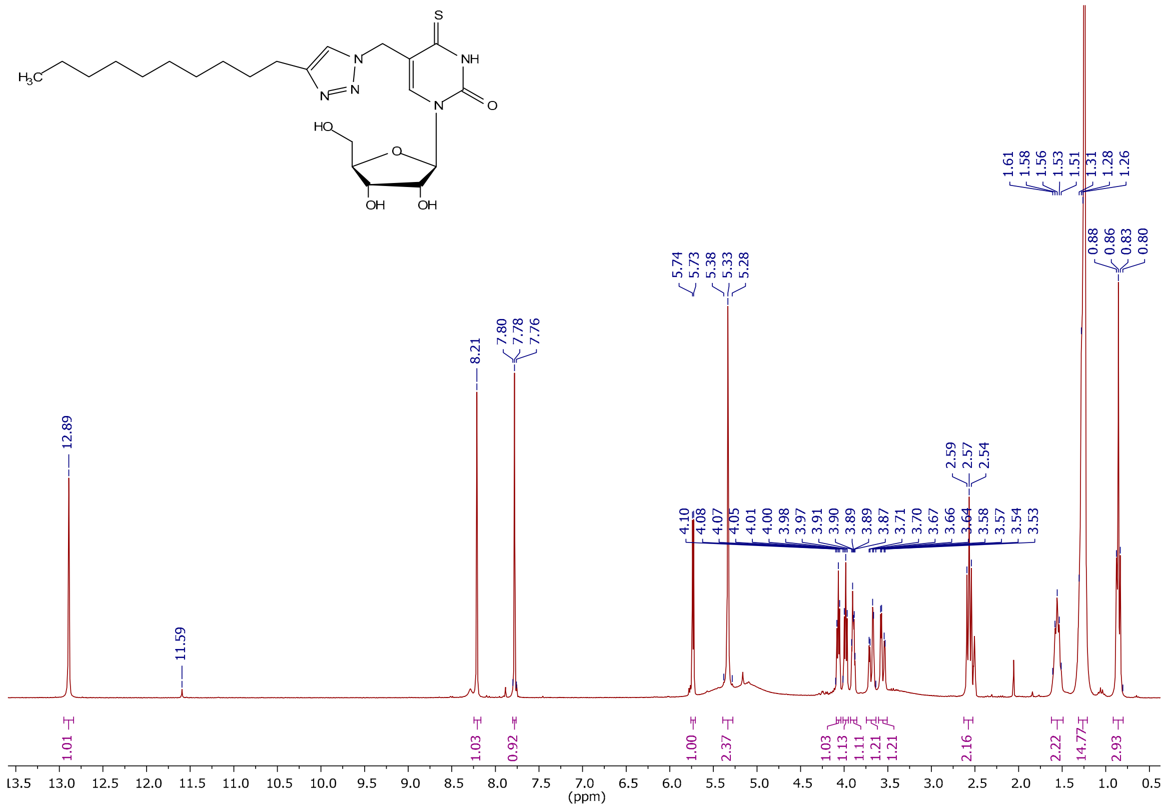
S
NH
O
N
N
N
N
H3C
HO
O
OH
OH
12.89
11.59
8.21
7.80
7.78
7.76
5.74
5.73
5.38
5.33
5.28
4.10
4.08
4.07
4.05
4.01
4.00
3.98
3.97
3.91
3.90
3.89
3.89
3.87
3.71
3.70
3.67
3.66
3.64
3.58
3.57
3.54
3.53
2.59
2.57
2.54
1.61
1.58
1.56
1.53
1.51
1.31
1.28
1.26
0.88
0.86
0.83
0.80
1.01
1.03
0.92
1.00
2.37
1.03
1.13
1.11
1.21
1.21
2.16
2.22
14.77
2.93
(ppm)

3g, ^{13}C

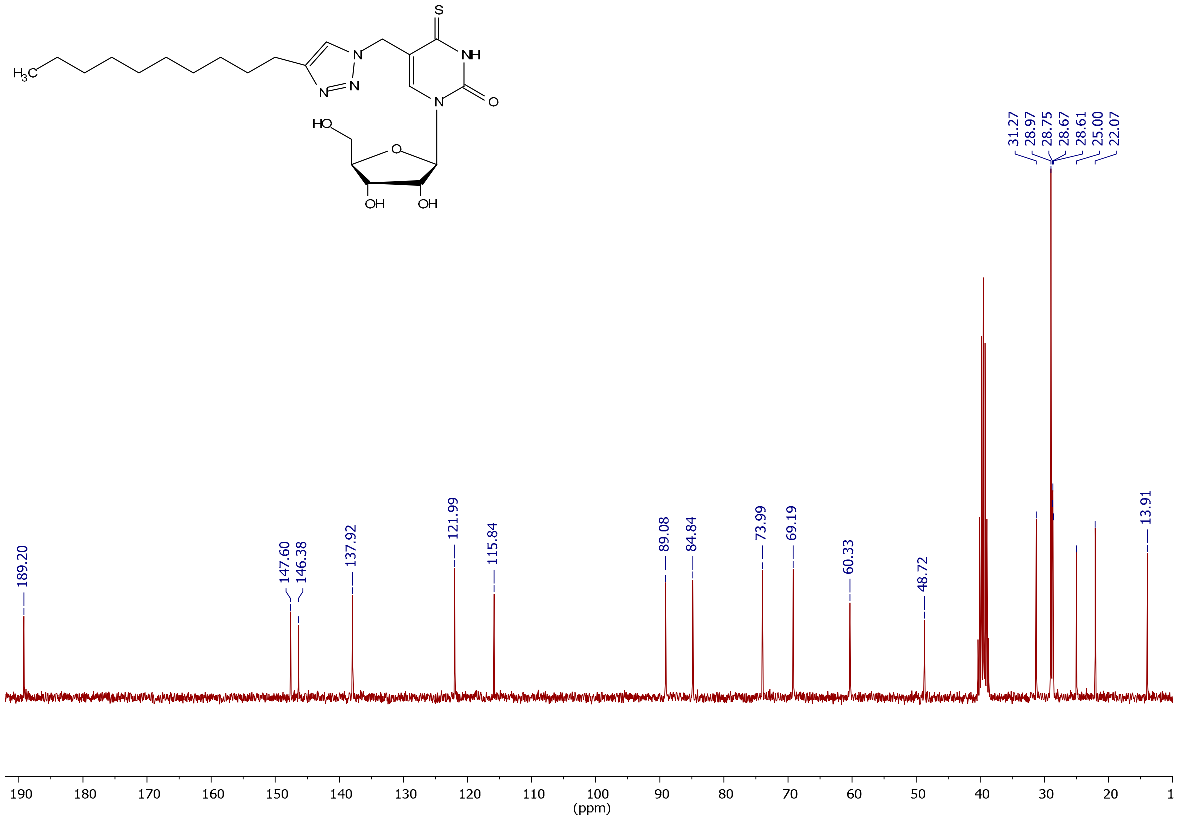
S
NH
O
N
N
N
N
H3C
HO
O
OH
OH
189.20
147.60
146.38
137.92
121.99
115.84
89.08
84.84
73.99
69.19
60.33
48.72
31.27
28.97
28.75
28.67
28.61
25.00
22.07
13.91
(ppm)

3h, ^{1}H

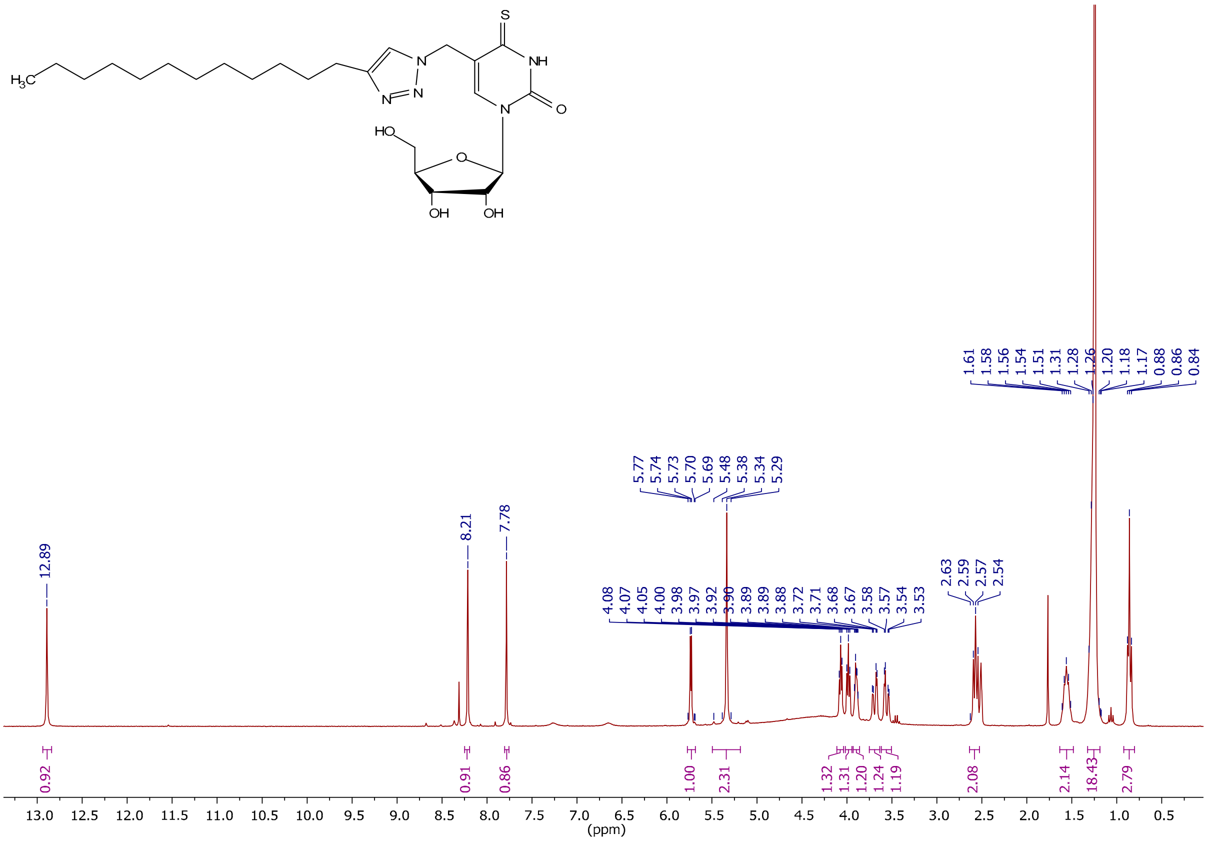

S
NH
O
N
N=N
N
H3C
HO
O
OH
OH
12.89
8.21
7.78
5.77
5.74
5.73
5.70
5.69
5.48
5.38
5.34
5.29
4.08
4.07
4.05
4.00
3.98
3.97
3.92
3.90
3.89
3.89
3.88
3.72
3.71
3.68
3.67
3.58
3.57
3.54
3.53
2.63
2.59
2.57
2.54
1.61
1.58
1.56
1.54
1.51
1.31
1.28
1.26
1.20
1.18
1.17
0.88
0.86
0.84
0.92
0.91
0.86
1.00
2.31
1.32
1.31
1.20
1.24
1.19
2.08
2.14
18.43
2.79
13.0 12.5 12.0 11.5 11.0 10.5 10.0 9.5 9.0 8.5 8.0 7.5 7.0 6.5 6.0 5.5 5.0 4.5 4.0 3.5 3.0 2.5 2.0 1.5 1.0 0.5
(ppm)

3h, ^{13}C

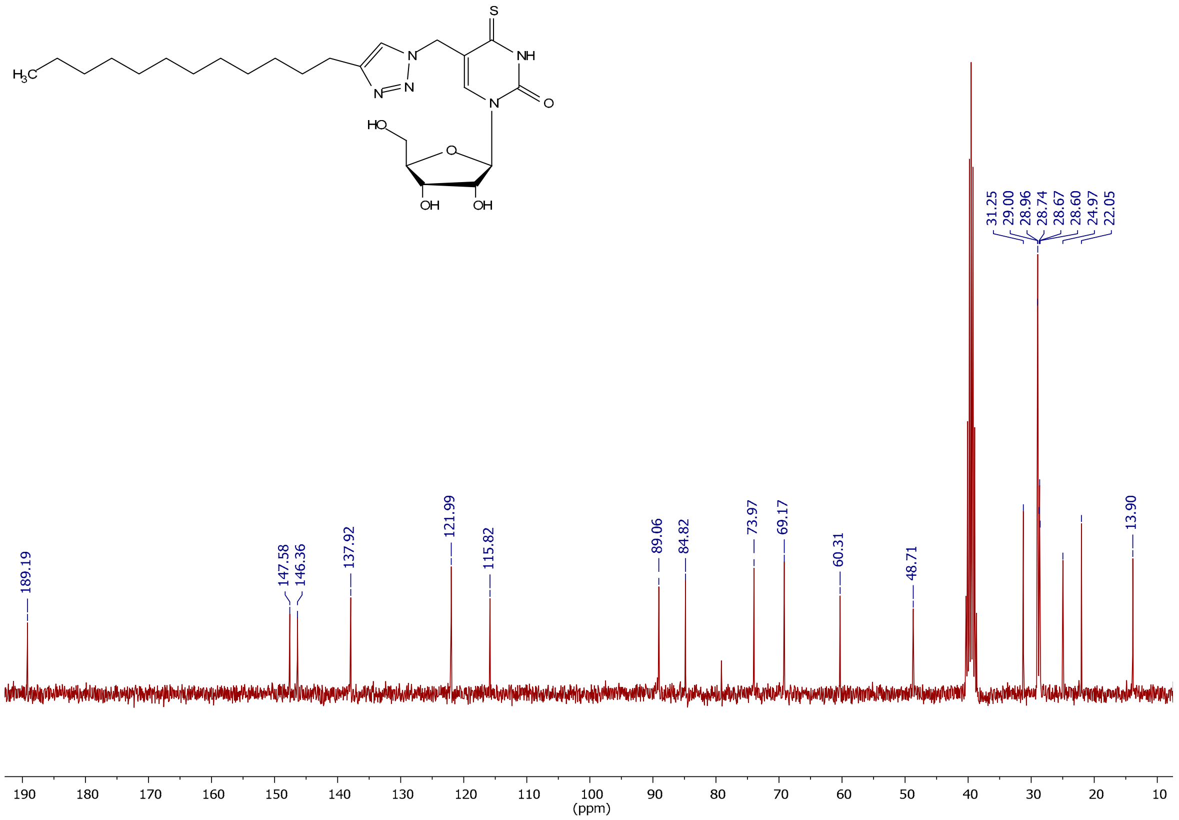

S
NH
O
N
N=N
N
H3C
HO
O
OH
OH
189.19
147.58
146.36
137.92
121.99
115.82
89.06
84.82
73.97
69.17
60.31
48.71
31.25
29.00
28.96
28.74
28.67
28.60
24.97
22.05
13.90
190 180 170 160 150 140 130 120 110 100 90 80 70 60 50 40 30 20 10
(ppm)

Mass-spectra of new compounds

3a

Display Report

Analysis Info

Analysis Name	D:\Data\Ikar\november\DM repeat 201124\DM 43 neg 201124_23_01_4871.d	Acquisition Date	11/20/2024 1:51:19 PM
Method	ik-negative-small full range.m	Operator	BDAL@DE
Sample Name	DM 43 neg 201124	Instrument	compact 8255754.20088
Comment			

Acquisition Parameter

Source Type	ESI	Ion Polarity	Negative	Set Nebulizer	0.4 Bar
Focus	Active	Set Capillary	3500 V	Set Dry Heater	180 °C
Scan Begin	50 m/z	Set End Plate Offset	-500 V	Set Dry Gas	4.0 l/min
Scan End	3000 m/z	Set Charging Voltage	2000 V	Set Divert Valve	Source
		Set Corona	0 nA	Set APCI Heater	0 °C

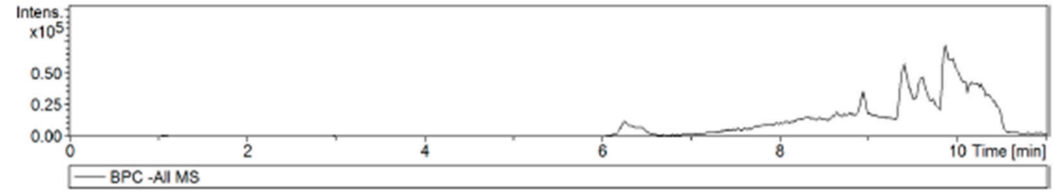

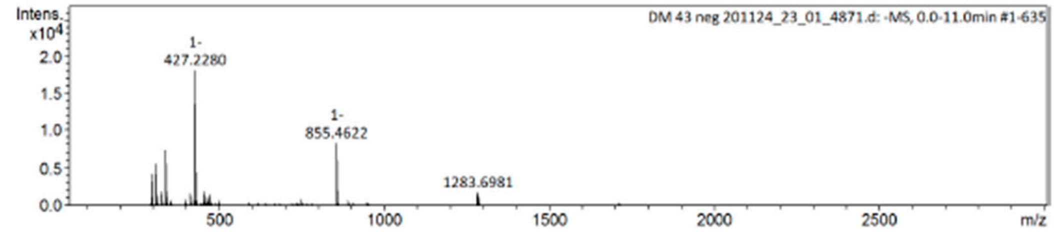

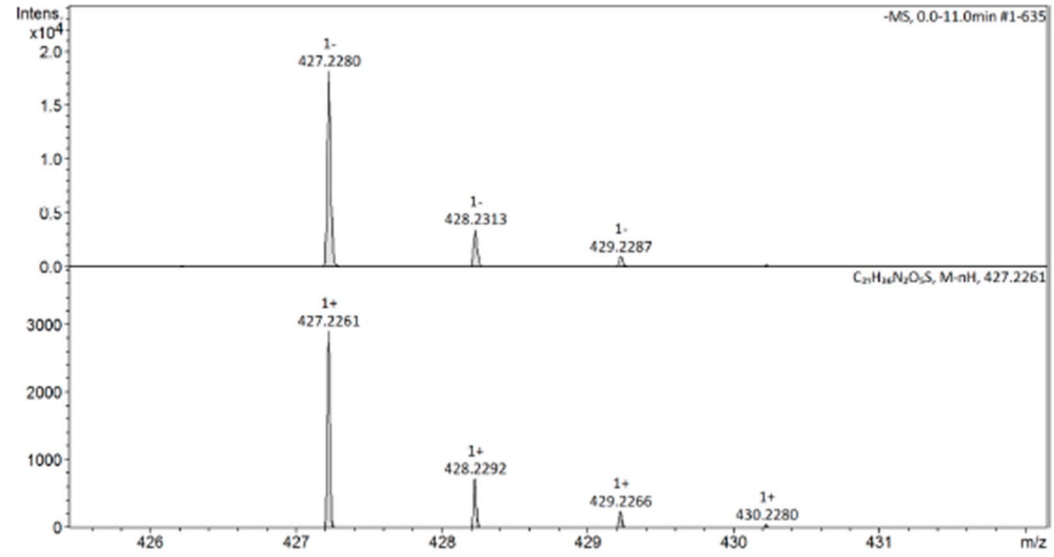

DM 43 neg 201124_23_01_4871.d

Bruker Compass DataAnalysis 4.3 printed: 4/23/2025 4:45:21 PM by: BDAL@DE Page 1 of 1
